# A randomized, double-blind, placebo-controlled study of the effect of ezetimibe on glucose metabolism in subjects with type 2 diabetes mellitus and hypercholesterolemia

**DOI:** 10.1186/s12944-015-0036-z

**Published:** 2015-05-01

**Authors:** Itori Saito, Kyoichi Azuma, Taro Kakikawa, Nobuyuki Oshima, Mary E Hanson, Andrew M Tershakovec

**Affiliations:** MSD KK, Japan development, Clinical research, Kitanomaru Square, 1-13-12 Kudan-kita, Chiyoda-ku, 102-8667 Tokyo Japan; Merck & Co., Inc, Kenilworth, NJ USA

**Keywords:** Fasting plasma glucose, Glycoalbumin, HbA1c, Japanese, LDL-C

## Abstract

**Background:**

Recent evidence points to an increased incidence of new-onset diabetes and a negative impact on glucose parameters with statin use. This study examined the safety of ezetimibe vs placebo for change from baseline to week 24 in HbA1c (primary endpoint), glycoalbumin, and fasting plasma glucose (secondary endpoints) in Japanese subjects with type 2 diabetes and hypercholesterolemia.

**Methods:**

This was a randomized, double-blind, placebo-controlled, parallel-group, multi-site trial. Adults with type 2 diabetes and hypercholesterolemia whose LDL-C measured <140 mg/dl (subjects receiving lipid-lowering drugs) or <160 mg/dl (subjects not receiving lipid-lowering drugs) at the start of the screening phase, were randomized after a 5-week wash-out period to ezetimibe 10 mg or placebo (1:1) for 24 weeks. Changes in HbA1c, glycoalbumin and fasting plasma glucose from baseline to week 24 were evaluated. The non-inferiority margin was set at 0.5% for HbA1c.

**Results:**

Overall, 152 subjects were randomized (75 to ezetimibe and 77 to placebo). From baseline to 24 weeks, HbA1c significantly increased in both the ezetimibe and placebo groups (between-treatment difference 0.08 [95% CI: −0.07 to 0.23]). Ezetimibe was statistically non-inferior to placebo. At 24 weeks, the mean change from baseline in glycoalbumin levels (between-treatment differences 0.00 [95% CI: −0.47, 0.47]) and fasting plasma glucose (between-treatment differences −4.8 [95% CI: −12.1, 2.1]) were similar in both treatment groups.

**Conclusions:**

These results suggest that ezetimibe 10 mg does not result in dysregulation of glucose metabolism in Japanese patients with type 2 diabetes and hypercholesterolemia over 24 weeks of treatment.

**Trial registration:**

ClinicalTrials.gov identifier NCT01611883.

**Electronic supplementary material:**

The online version of this article (doi:10.1186/s12944-015-0036-z) contains supplementary material, which is available to authorized users.

## Background

While diabetes, hypertension and hypercholesterolemia are each independent risk factors for coronary artery disease (CAD), concomitant occurrence of these factors may lead to a cumulative increase in CAD risk [1]. Management of these risk factors is important for reducing the risk of atherosclerosis and the risk of ischemic heart disease and CAD. In addition to lipid management and blood pressure control, glycemic control is a basic component in the management of diabetes and is achieved in part via diabetes self-management, education, exercise and improved diet, the latter elements being the cornerstones of treatment for diabetes and lipids. As such, it is important that glucose metabolism not be adversely affected when patients take lipid-lowering treatment.

Post-hoc analyses of large clinical trials and meta-analyses have demonstrated a dose- and potency-dependent effect for increased incidence of new-onset diabetes and mild elevation of hemoglobin A1c (HbA1c) and fasting plasma glucose with statin use [2-7]. However, it was determined that the benefits of reducing the risk of cardiovascular events with statin use significantly outweigh the potential risks of worsening glycemic control or developing new-onset diabetes [8]. Ezetimibe is an inhibitor of a small intestine cholesterol transporter. It lowers low-density lipoprotein cholesterol (LDL-C) by 15-20% through inhibition of exogenous and biliary cholesterol absorption in the digestive tract by inhibiting the Niemann-Pick C1-Like 1 in the brush border of enterocytes [9,10]. A significant reduction in insulin resistance and/or fatty liver have been reported in small clinical trials using the combination of pravastatin with ezetimibe [11] or ezetimibe monotherapy [12,13]. However, other small studies have observed no changes in parameters of glucose metabolism and insulin sensitivity with ezetimibe monotherapy [14-16]. Limited data suggest that ezetimibe, whether added to a statin or used as a monotherapy, does not have an adverse effect on glucose metabolism [17,18]. However, in light of recent findings regarding the increased incidence of new-onset diabetes and the impact on glucose parameters with the use of statins [2-7], it is important to assess any potential for these same issues with ezetimibe use. This study was conducted to prospectively examine the effects of ezetimibe on glucose metabolism in patients with type 2 diabetes and hypercholesterolemia.

The primary objective of this study was to examine the safety of ezetimibe compared with placebo with regard to change in HbA1c from baseline to week 24 in subjects with type 2 diabetes and hypercholesterolemia. Secondary objectives included comparing ezetimibe to placebo in subjects with type 2 diabetes and hypercholesterolemia for change in glycoalbumin and fasting plasma glucose from baseline to week 24; the proportion of patients having onset of “exacerbation of diabetes mellitus” (assessed by index of blood glucose control, changes in diabetes medications, and compliance to diet and exercise therapy); the proportion of patients with changes to diabetes medications due to worsening of diabetes.

## Methods

### Study design

This was a randomized, double-blind, placebo-controlled, parallel-group, multi-site trial (protocol number: PN367, Additional file 1; ClinicalTrials.gov identifier NCT01611883) of ezetimibe in subjects with type 2 diabetes and hypercholesterolemia carried out at 19 sites in Japan and conducted in conformance with Good Clinical Practice and the Declaration of Helsinki between August 2012 and January 2014. The protocol was approved by the institutional review boards for each study site (Japanese Association for the Promotion of State of the Art in Medicine - The Second Institutional Review Board; Tokushukai Group Institutional Review Board; Hirokuni Hospital Institutional Review Board; Doujin Memorial Foundation Meiwa Hospital Institutional Review Board; Clinical Research Promotion Network Japan Institutional Review Board; Hatamoto Institutional Review Board). All patients provided written, informed consent prior to entering the study. Each subject participated in the trial for approximately 33 weeks from the time the subject signed the informed consent form through the final contact. After screening, eligible subjects stopped any current lipid-lowering medications for 5 weeks prior to receiving assigned treatment for approximately 24 weeks. Ezetimibe 10 mg or placebo was administered orally once daily and follow-up visits occurred at 4, 12, 20 and 24 weeks of treatment. Serious adverse events were monitored from the time informed consent was obtained until 30 days after the administration of the last dose of study drug.

#### Subjects

Subjects with a diagnosis of type 2 diabetes mellitus and hypercholesterolemia aged 20 to 75 years were selected to participate in the trial, including those undergoing treatment with oral anti-diabetic drugs or insulin or both, and who had no change in the type, dose, and regimen of drugs for the treatment of diabetes within 12 weeks before the start of the screening phase. However, small changes in insulin dosing +/−5U were acceptable. Subjects undergoing diet and exercise therapy with no change in either therapy within 4 weeks before the start of the screening phase (however, exercise therapy was not necessarily applicable in the case of patients with coexisting conditions judged not appropriate to meet this criterion). Subjects were included whose LDL-C measured at the start of the screening phase < 140 mg/dl if they had been receiving lipid lowering drugs; in subjects who had not been receiving lipid lowering drugs, LDL-C measures < 160 mg/dl were required. At 1 week prior to randomization, all subjects were required to have LDL-C level ≥ 120 mg/dl and < 160 mg/dl in order to participate in the study. Subjects were excluded if their triglyceride value exceeded 400 mg/dl, HbA1c value was ≥8.4%, (HbA1c was recorded in the National Glycohemoglobin Standardization Program [NGSP]), and/or fasting plasma glucose was ≥170 mg/dl at screening or 1 week prior to randomization. Subjects were excluded if they had history of coronary heart disease (CHD), stroke, or arteriosclerosis obliterans; if they had hypercholesterolemia associated with hypothyroidism; or if they had active or severe hepatic disease. Concomitant therapy with other lipid modifying therapies including statins, fibrates, niacin, cyclosporine, systemic corticosteroids, and investigational drugs was prohibited.

### Safety and efficacy endpoints

The primary endpoint of this study was to examine the safety of ezetimibe compared with placebo with regard to change in HbA1c from baseline to week 24. Secondary endpoints included comparing ezetimibe to placebo for change in glycoalbumin and fasting plasma glucose from baseline to week 24. Additionally, the proportion of subjects having exacerbation of diabetes mellitus (exacerbation of diabetes mellitus was judged by the investigator taking into account the blood glucose control indices, diabetes medications, and compliance to diet and exercise therapy) and the proportion of subjects with changes in diabetes medication due to worsening of diabetes was calculated (small changes in insulin dosing ±5U were excluded). Additional safety was monitored through collection of adverse events (AEs), adverse drug reactions, hematology (white blood cell count, red blood cell count, Hb, hematocrit, platelet count), biochemistry (aspartate aminotransferase, alanine aminotransferase, gamma glutamyl transpeptidase, alkaline phosphatase, lactate dehydrogenase, creatine phosphokinase, total bilirubin, direct bilirubin, total protein, blood urea nitrogen, uric acid, creatinine, Na, K, Cl) and vital signs (body weight, blood pressure, pulse) throughout the study. For efficacy endpoints the percent change from baseline to week 24 in serum lipids, including LDL-C, estimated by the Friedewald equation, total cholesterol, triglycerides, and high-density lipoprotein cholesterol (HDL-C) were measured. Non-HDL-C was calculated by subtracting HDL-C from total cholesterol. All laboratory measurements, including glucose metabolism and lipid parameters, were conducted at a central laboratory.

### Statistical analysis

One-hundred-forty eight subjects were planned for enrollment with at least 45 (30%) receiving insulin. For the primary safety hypothesis of this study, the difference between the ezetimibe and placebo groups in the least squares mean change in HbA1c from baseline to 24 weeks after treatment was assumed to be 0.1%, with standard deviation of 0.7% and a non-inferiority margin of 0.5%. The standard deviation and between groups difference were determined using a previous open-label study (a Phase III, trial in diabetic mellitus subjects with hypercholesterolemia) which provided a limited number of patients. The non-inferiority margin was given as a clinically meaningful exacerbation of diabetes control in medical practice. With this hypothesis, the number of subjects per group was projected to be 66 with a two-sided 5% (one-sided 2.5%) probability of type 1 error, 90% power, and 1:1 allocation to each of the groups. The sample size was set to 148 to take into account a 10% dropout rate. There is only one primary hypothesis for this study; therefore, the issue of multiplicity will not occur.

The data set for the statistical analysis of safety is the per protocol set (PPS). Missing values were not replaced with other values. The full analysis set (FAS) was used for the sensitivity analysis of the primary trial objective and was defined as all randomized subjects meeting the inclusion criteria who received study drug and for whom a baseline and at least one post-baseline measurement was obtained. This was the data set for the efficacy analysis. Missing values were not replaced with other values. The PPS was defined as all randomized subjects meeting inclusion criteria who were not excluded from the FAS and were at least 75% compliant with study medication. The all subjects as treated (ASaT) group was defined as all subjects who took at least one dose of study drug during the treatment period. The ASaT was used for the analysis of AEs.

The longitudinal analysis of covariance was used to assess the primary (HbA1c) and secondary safety endpoints (glycoalbumin and fasting plasma glucose). Baseline, treatment group, HbA1c (2 categories: < 7.4% and ≥7.4% to < 8.4%), insulin use (2 categories: yes, no), time, and “time × treatment group” interaction were included in the model. So as not to place restriction on the time-course curve of mean values, time was a categorical variable. Between-group difference in mean amount of change from baseline at measurement time points was estimated and tested. Correlation between time-course measured values was modeled using an unstructured covariance matrix, but if this calculation did not converge, Toeplitz was used for correlation. Based on the above unstructured model, the difference in least squares (LS) mean from the placebo group and the 95% confidence interval (CI) were calculated. The LS mean value of the amount of change from baseline with each group and the 95% CI were also calculated. As sensitivity analysis of the primary endpoint, the same analysis was conducted with the FAS. A between-group comparison was conducted using Fisher’s exact test for the proportion of subjects with “exacerbation of diabetes”. A between-group comparison using Fisher’s exact test was also conducted for the proportion of subjects with changes in diabetes medications due to worsening of diabetes. Summary statistics with standard deviations were calculated for demographic and baseline characteristics and the number of subjects with AEs and the incidence of AEs were summarized by treatment group and by system organ class and event. The percent change from baseline to each measurement time point was calculated by treatment group for lipid parameters using the same longitudinal analysis of covariance used for the primary safety endpoint. Lipids included LDL-C, total cholesterol, triglycerides, HDL-C, and non-HDL-C. No adjustments were made for multiplicity.

## Results

**Figure 1:**
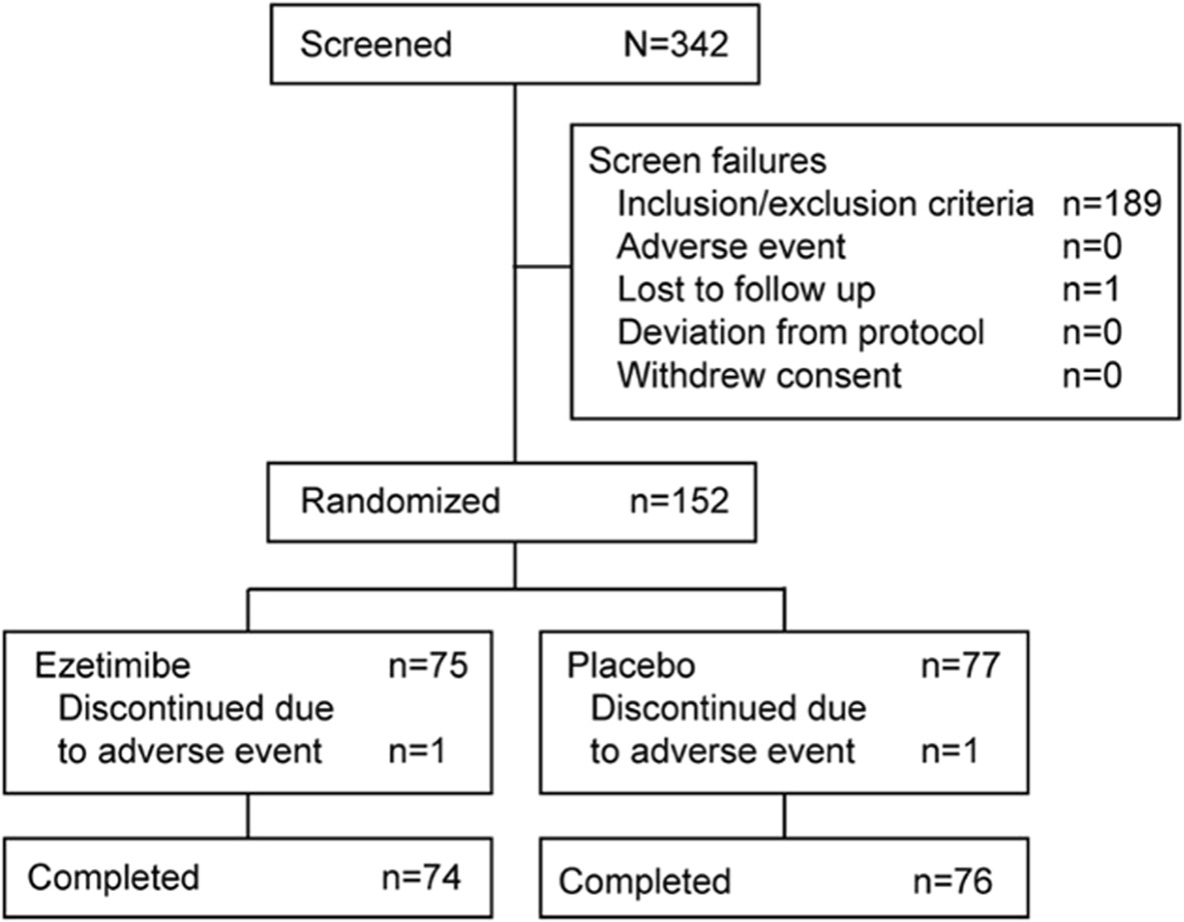
Patient flow through the study.

A total of 342 subjects were screened, resulting in 152 randomized to treatment (75 to ezetimibe 10 mg and 77 to placebo; Figure 1). In each treatment group, only 1 subject discontinued due to an AE. No patients were excluded from the safety and efficacy analyses. Baseline demographics and clinical characteristics were similar between treatment groups (Table 1). Approximately 2/3 of subjects were male, the mean (± standard deviation [SD]) age was 60 (±10) years, with a mean (± SD) body mass index of 26 (±5) kg/m^2^. The vast majority (95%) of subjects had coexisting diseases. There was no notable difference observed between the two groups in coexisting diseases or the use of concomitant medications (Table 1). At baseline mean HbA1c was 7.0%, glycoalbumin was 17.3%, and fasting plasma glucose was 126.3 mg/dL.

**Table 1:** **Baseline demographics and clinical characteristics**

	**Ezetimibe**	**Placebo**	**Total**
Subjects in population	75	77	152
Male, n (%)	46 (61.3)	48 (62.3)	94 (61.8)
Mean age, yrs (SD)	59.3 (10.8)	60.0 (9.7)	59.6 (10.2)
Mean body mass index, kg/m2 (SD)	26.2 (5.3)	25.7 (3.9)	25.9 (4.6)
Coexisting diseases, yes; n (%)	70 (93.3)	74 (96.1)	144 (94.7)
Selected coexisting diseases, n (%):			
Cardiac disease other than CHD	3 (4.0)	4 (5.2)	7 (4.6)
Hypertension	37 (49.3)	35 (45.5)	72 (47.4)
Hepatic steatosis	24 (32.0)	26 (33.8)	50 (32.9)
Hyperuricaemia	6 (8.0)	7 (9.1)	13 (8.6)
Obesity	2 (2.7)	1 (1.3)	3 (2.0)
Concomitant medications, yes; n (%)	75 (100)	77 (100)	152 (100)
Selected concomitant medications, n (%):			
Oral anti diabetes agents	70 (93.3)	72 (93.5)	142 (93.4)
Dipeptidyl peptidase-4 inhibitors	39 (52.0)	29 (37.7)	68 (44.7)
Sulfonylureas	27 (36.0)	22 (28.6)	49 (32.2)
Glinides	3 ( 4.0)	10 (13.0)	13 (8.6)
Thiazolidine	12 (16.0)	9 (11.7)	21 (13.8)
Biguanides	34 (45.3)	28 (36.4)	62 (40.8)
Alpha-glucosidase inhibitors	24 (32.0)	24 (31.2)	48 (31.6)
Thiazolidine + Biguanides	1 (1.3)	-	1 (0.7)
Glinides + Alpha-glucosidase inhibitors	2 (2.7)	-	2 (1.3)
Insulin administration yes; n (%)	23 (30.7)	24 (31.2)	47 (30.9)
Hypertension agents	35 (46.7)	30 (39.0)	65 (42.8)
Calcium channel blocker	19 (25.3)	15 (19.5)	34 (22.4)
Beta blocker	2 (2.7)	1 (1.3)	3 (2.0)
Alpha blocker	5 (6.7)	2 (2.6)	7 (4.6)
ARB	22 (29.3)	17 (22.1)	39 (25.7)
Diuretic	3 (4.0)	1 (1.3)	4 (2.6)
ACE inhibitor	2 (2.7)	4 (5.2)	6 (3.9)
ARB + Diuretic	3 (4.0)	1 (1.3)	4 (2.6)
ARB + Calcium channel blocker	3 (4.0)	3 (3.9)	6 (3.9)
Mean duration of diabetes, months (SD)	88.9 (74.0)	94.9 (93.8)	92.0 (84.4)
Mean HbA1c, % (SD)	7.0 (0.6)	7.0 (0.6)	7.0 (0.6)
<7.4%, n (%)	56 (74.7)	58 (75.3)	114 (75.0)
≥7.4%, <8.4%, n (%)	19 (25.3)	19 (24.7)	38 (25.0)
Mean glycoalbumin, % (SD)	17.3 (2.3)	17.4 (2.6)	17.3 (2.4)
Mean fasting plasma glucose, mg/dL (SD)	125.7 (22.2)	126.9 (17.3)	126.3 (19.8)
Mean LDL-C, mg/dL (SD)	138.6 (11.2)	139.4 (10.4)	139.0 (10.8)
Mean total cholesterol, mg/dL (SD)	217.6 (16.2)	219.0 (21.1)	218.3 (18.8)
Mean triglycerides, mg/dL (SD)	119.6 (57.3)	129.5 (63.8)	124.6 (60.7)
Mean HDL-C, mg/dL (SD)	55.0 (13.5)	53.7 (12.8)	54.3 (13.1)
Mean non-HDL-C, mg/dL (SD)	162.6 (15.2)	165.3 (16.4)	164.0 (15.9)

ARB = angiotensin receptor blocker; ACE = angiotensin converting enzyme; HbA1c = hemoglobin A1c HDL-C = high-density lipoprotein cholesterol; LDL-C = low-density lipoprotein cholesterol.

**Figure 2:**
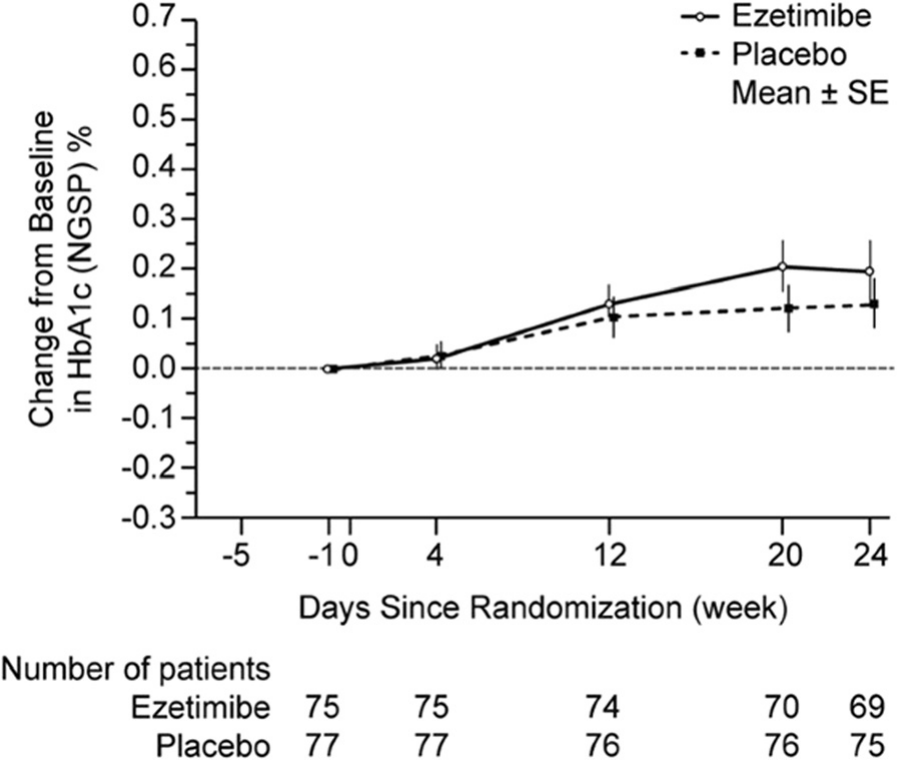
Change from baseline in HbA1c (%) over 24 weeks of treatment with ezetimibe 10 mg or placebo (PPS).

The mean baseline values and change from baseline in blood glucose control indices in the ezetimibe 10 mg and placebo groups are shown in Table 2. Over the course of the 24 weeks of treatment, HbA1c significantly increased from baseline in both the ezetimibe 10 mg and placebo groups (Figure 2). At 24 weeks, the least-squares (LS) mean (95% CIs) changes from baseline in HbA1c were 0.22 (0.11 to 0.34) and 0.14 (0.03 to 0.25) for the ezetimibe 10 mg and placebo groups, respectively (Table 2). The between-treatment difference in the change from baseline was 0.08 (−0.07 to 0.23). The ezetimibe 10 mg group was statistically non-inferior to the placebo group, compared with the pre-defined non-inferiority margin of 0.5 (Table 2). The sensitivity analysis was consistent with the primary results, demonstrating non-inferiority of ezetimibe. Results were generally consistent across subgroups defined by baseline HbA1c level (<7.4%, vs. ≥7.4% to <8.4%) and by insulin administration at baseline (yes vs. no; data not shown).

**Table 2:** **Change from baseline to 24 weeks in glucose parameters**

		**Baseline**	**Week 24**	**Change**		
	**Treatment (n)**	**Mean (SE)**	**Mean (SE)**	**Mean (SE)**	**LS Mean (95% CI)**	**P-value**
HbA1c (%)	Ezetimibe (n = 69)	6.94 (0.07)	7.14 (0.10)	0.20 (0.06)	0.22 (0.11, 0.34)	<0.001
	Placebo (n = 75)	6.96 (0.06)	7.09 (0.07)	0.13 (0.05	0.14 (0.03, 0.25)	0.015
	Ezetimibe vs Placebo			Difference in LS mean (95% CI)	0.08 (−0.07, 0.23)	0.281
Glyocalbumin (%)	Ezetimibe (n = 69)	17.16 (0.26)	17.14 (0.30)	−0.02 (0.17)	−0.02 (−0.37, 0.34)	0.919
	Placebo (n = 75)	17.21 (0.27)	17.20 (0.29)	−0.01 (0.17)	−0.02 (−0.37, 0.33)	0.918
	Ezetimibe vs Placebo			Difference in LS mean (95% CI)	0.00 (−0.47, 0.47)	1.000
Fasting plasma glucose (mg/dL)	Ezetimibe (n = 69)	124.7 (2.6)	128.0 (2.8)	3.3 (3.0)	6.6 (1.1, 12.1)	0.019
	Placebo (n = 75)	126.7 (2.0)	135.4 (2.9)	8.7 (2.5)	11.4 (6.1, 16.7)	<0.001
	Ezetimibe vs Placebo			Difference in LS mean (95% CI)	−4.8 (−12.1, 2.5)	0.194

**Figure 3:**
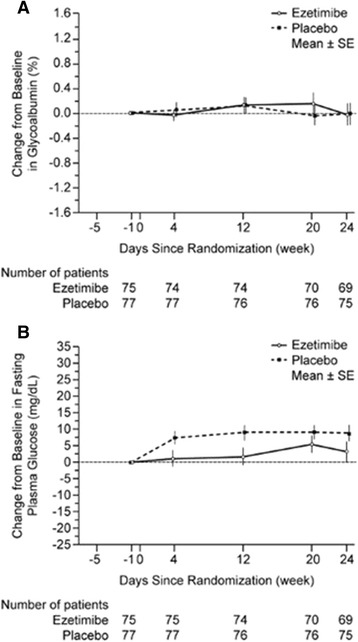
Change from baseline in **A)** glycoalbumin (%) and **B)** fasting plasma glucose (mg/dL) over 24 weeks of treatment with ezetimibe 10 mg or placebo (PPS).

At 24 weeks, the LS mean changes (95% CIs) from baseline in glycoalbumin were −0.02 (−0.37, 0.34) and −0.02 (−0.37, 0.33) for the ezetimibe 10 mg and placebo groups, respectively (Table 2). Over the course of the 24 weeks of treatment, changes from baseline in glycoalbumin levels in both treatment groups were similar (Figure 3). The between-treatment difference in the change from baseline at 24 weeks was (0.00; 95% CI: −0.47, 0.47; Table 2).

**Figure 4:**
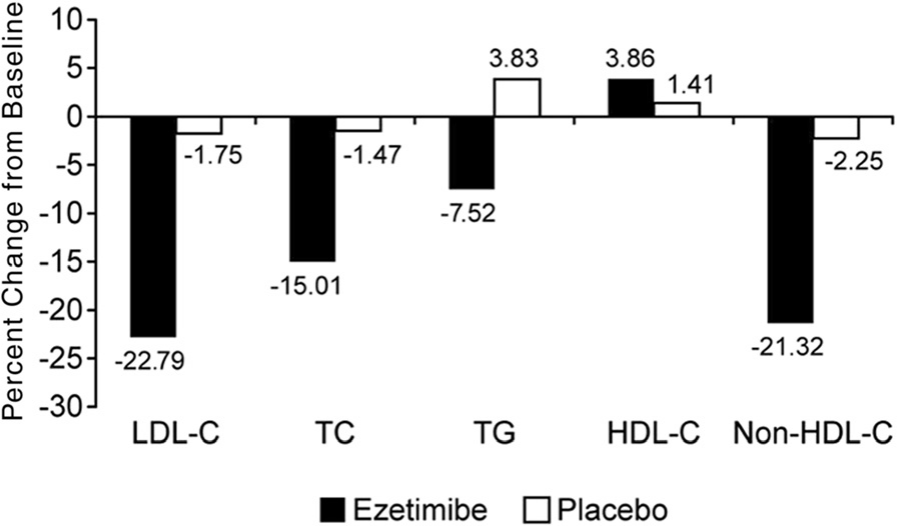
Percent change from baseline to 24 weeks in lipid parameters (FAS). No patient had triglyceride level >400 mg/dL after allocation.

At 24 weeks, the LS mean changes (95% CIs) from baseline in fasting plasma glucose were 6.6 (1.1, 12.1) and 11.4 (6.1, 16.7) for the ezetimibe 10 mg and placebo groups, respectively (Table 2). Over the course of the 24 weeks of treatment, fasting plasma glucose significantly increased from baseline in both treatment groups (Figure 4). The between-treatment difference in the change from baseline at 24 weeks was (−4.8; 95% CI: −12.1, 2.1; Table 2).

There was no significant difference in exacerbation of diabetes between treatment groups (p = 0.78). Seven subjects (9%) in the ezetimibe group and 6 subjects (8%) in the placebo group experienced exacerbation of diabetes. Similarly, there was no significant difference between groups in the proportion of subjects with changes to diabetes medications due to worsening of diabetes (p = 0.37). Seven subjects (9%) in the ezetimibe group and 4 subjects (5%) in the placebo group made changes to their diabetes medications due to worsening of their diabetes during the study period.

The percent changes from baseline to 24 weeks in LDL-C, total cholesterol, triglycerides, HDL-C and non-HDL-C are shown in Figure 4. After 24 weeks of treatment, the change from baseline was statistically significant for all 5 lipid parameters in subjects treated with ezetimibe 10 mg (P < 0.05) but was not statistically significant in subjects who received placebo. The percent differences (95% CI) between treatment groups (ezetimibe vs. placebo) at 24 weeks were statistically significant except for HDL-C: LDL-C: −21.05% (−25.06, −17.03) P < 0.001; total cholesterol: −13.54% (−16.66, −10.42) P < 0.001; HDL-C: 2.45% (−1.71, 6.61) P = 0.25; triglycerides: −11.36% (−21.27, −1.44) P = 0.03; and non-HDL-C: −19.07% (−22.71, −15.43) P < 0.001.

A summary of AEs for the ASaT population is shown in Table 3. Approximately half (49.3%) of subjects treated with ezetimibe 10 mg reported experiencing an AE and nearly 2/3 (64.9%) of subjects receiving placebo reported experiencing an AE during the study period. Three subjects (4.0%) in the ezetimibe 10 mg group and 1 subject (1.3%) in the placebo group reported a drug-related AE. There were 2 subjects (2.7%) in the ezetimibe 10 mg group (1 cataract and 1 abdominal pain) and 5 subjects (6.5%) in the placebo group (2 cases of angina—one prior to study participation and one after study completion; 1 accidental overdose, 1 ankle fracture, 1 spinal osteoarthritis) that reported serious AEs. None of these AEs were considered related to study drug, nor were there any deaths in either group. The incidence of AEs of interest was generally similar between treatment groups. No serious AEs, deaths or AEs leading to study discontinuation occurred in this study.

**Table 3:** **Summary of adverse events**

	**Ezetimibe**	**Placebo**
	**n (%)**	**n (%)**
Subjects in population	75	77
With ≥1 adverse events	37 (49.3)	50 (64.9)
With drug-related adverse events	3 (4)	1 (1.3)
With serious adverse events	2 (2.7)	5 (6.5)
Discontinued due to an adverse event	1 (1.3)	1 (1.3)
Discontinued due to a serious adverse event	0 (0)	1 (1.3)

## Discussion

The results of this study showed that the ezetimibe 10 mg group was statistically non-inferior to the placebo group with respect to change in HbA1c. Over the course of the 24 weeks of treatment, changes from baseline in glycoalbumin and fasting plasma glucose levels in both treatment groups were similar. In addition, differences in exacerbation of diabetes or changes to patients’ diabetes medications due to worsening of their diabetes during the study period did not change significantly. However, differences in change from baseline lipid parameters between ezetimibe and placebo were statistically significant at 24 weeks, except for HDL-C. Ezetimibe was generally safe and well-tolerated.

Results of 2 meta-analyses suggested that treatment with statins may be associated with a small (9%) increased risk for developing diabetes [4,19]. This was based on measures of glucose regulation such as HbA1c, and fasting plasma glucose. However, published information regarding the incident relationship of ezetimibe treatment to glucose regulations and new onset diabetes is limited. Trials that did report on glucose changes with ezetimibe treatment included subjects with diabetes or metabolic syndrome, both populations that may have impaired glucose function at study start [20,21]. Therefore, it is difficult to fully assess the individual impact of ezetimibe on glucose metabolism based on the published data.

Several studies in animals have suggested that ezetimibe may have positive effects on glycemic control, including reduced weight gain, diet-induced hyperglycemia and insulin resistance [22], improvement in insulin and plasma glucose response in obese fatty rats [23], and improvement in glucose tolerance, increased insulin sensitivity, and protecting the function of beta-cells in diabetic mice [24]. In addition, one clinical trial showed that after 12 weeks of treatment with ezetimibe 10 mg monotherapy, reductions in HbA1c and fasting plasma glucose were seen in Japanese patients with insulin resistance [25]. These results are consistent with those of the current trial, suggesting that ezetimibe does not confer an increased risk of glucose dysregulation. However, in the previous studies that observed neutral changes in parameters of glucose metabolism and insulin sensitivity with ezetimibe monotherapy, the study populations were small, limiting the generalizability of the results to larger populations [14-16]. More recent preliminary data from two post-hoc, pooled analyses in subjects without diabetes suggest that ezetimibe monotherapy or ezetimibe added to statin does not have a negative effect on glucose metabolism [17,18].

The magnitude of change from baseline in lipids, including LDL-C, total cholesterol, triglycerides and HDL-C, was consistent with those demonstrated in previous 12-week trials of ezetimibe monotherapy vs. placebo [26-33]. A meta-analysis of these same trials demonstrated that ezetimibe monotherapy conferred significant reductions in LDL-C, triglyceride, and total cholesterol levels as well as a significant increase in HDL-C level compared with placebo [34]. Except for the significant HDL-C increases, the results of the meta-analysis are consistent with those of the current trial in Japanese patients.

Ezetimibe 10 mg was generally well tolerated. Most AEs were mild in intensity with few related to treatment. There were no unexpected AEs reported based on what has been observed in other clinical trials with ezetimibe monotherapy [34].

This study had some limitations and the results must be interpreted with caution. The population was limited to Japanese patients and may not be generalizable to other populations. The short-term safety profile of ezetimibe monotherapy appears to be similar to placebo. This short-term study limits the ability to extrapolate the results to long-term profiles. However, the recently completed IMPROVE-IT trial assessed the incremental cardiovascular benefit of LDL-C lowering over 7 years with ezetimibe 10 mg added to simvastatin (mainly 40 mg) compared with simvastatin monotherapy in patients presenting with acute coronary syndromes [35-37]. The study investigators reported that the trial met its primary and secondary composite efficacy endpoints.

## Conclusions

In conclusion, these data suggest that treatment with ezetimibe 10 mg does not result in dysregulation of glucose metabolism over 24 weeks in Japanese patients with type 2 diabetes and hypercholesterolemia. Ezetimibe was generally well-tolerated with reduction of atherogenic lipid parameters.

## Additional file

Additional file 1:
**Redacted trial protocol for study 367 entitled: Examination of the effect of ezetimibe on glucose metabolism: Randomized, double-blind, placebo-controlled study in type 2 diabetes mellitus patients with hypercholesterolemia - Phase 4.**

## Abbreviations

AEs: Adverse events
ASaT: All subjects as treated
CAD: Coronary artery disease
CI: Confidence interval
FAS: Full analysis set
HbA1c: Hemoglobin A1c
HDL-C: High-density lipoprotein cholesterol
LDL-C: Low-density lipoprotein cholesterol
LS: Least squares
PPS: Per protocol set
