# Supplementary material for: A randomized, double-blind, placebo-controlled study of the effect of ezetimibe on glucose metabolism in subjects with type 2 diabetes mellitus and hypercholesterolemia
Source: Lipids Health Dis. 2015 May 1;14:40. doi: 10.1186/s12944-015-0036-z (PMC4450465; doi:10.1186/s12944-015-0036-z)
Supplement: Additional file 1: — Redacted trial protocol for study 367 entitled: Examination of the effect of ezetimibe on glucose metabolism: Randomized, double-blind, placebo-controlled study in type 2 diabetes mellitus patients with hypercholesterolemia - Phase 4. [file 12944_2015_36_MOESM1_ESM.pdf]

MERCK

Merck's policy on posting of redacted study protocols on journal websites is described in Merck Guidelines for Publication of Clinical Trials in the Scientific Literature on the www.merck.com website.

For publicly posted protocols, Merck redacts the background and rationale sections because these sections may contain proprietary information. Merck also redacts the names of any individuals due to privacy issues. The appendices generally are not provided because they may be lengthy and contain non-essential information. The publicly posted protocol includes all the key sections that are relevant to evaluating the study, specifically those sections describing the study objectives and hypotheses, the patient inclusion and exclusion criteria, the study design and procedures, the efficacy and safety measures, the statistical analysis plan, and amendments relating to those sections.

This report may include approved and non-approved uses, formulations, or treatment regimens. The results reported may not reflect the overall profile of a product. Before prescribing any product mentioned this report, healthcare professionals should consult local prescribing information for the product approved in their country.

Copyright © 2014 Merck Sharp & Dohme Corp., a subsidiary of Merck & Co., Inc.
All Rights Reserved. Not for regulatory or commercial use.

1. TITLE PAGE

Title	Examination of the effect of ezetimibe on glucose metabolism<br>- Randomized, double-blind, placebo-controlled study in type 2 diabetes mellitus patients with hypercholesterolemia -<br>Phase4, Protocol No. 367 (also known as SCH 58235, P06541)
Sponsor	Merck Sharp & Dohme Corp., a subsidiary of Merck & Co., Inc.
Sponsor's Address	KITANOMARU SQUARE 1-13-12 Kudan-kita Chiyoda-ku, Tokyo, 102-8667
Clinical Monitor	Redacted
Doc ID	Not Applicable
Phase	4
Date of Finalization of This Current Version of the Protocol	15 Mar 2012 Final (Ver.00)
Protocol Template Approval Date	8 JUN 2011

C O N F I D E N T I A L

TRIAL PROTOCOL

THIS CONFIDENTIAL INFORMATION ABOUT AN INVESTIGATIONAL DRUG OR PRODUCT IS PROVIDED FOR THE EXCLUSIVE USE OF INVESTIGATORS OF THIS DRUG OR PRODUCT AND IS SUBJECT TO RECALL AT ANY TIME. THE INFORMATION IN THIS DOCUMENT MAY NOT BE DISCLOSED UNLESS SUCH DISCLOSURE IS REQUIRED BY APPLICABLE LAW OR REGULATIONS. SUBJECT TO THE FOREGOING, THIS INFORMATION MAY BE DISCLOSED ONLY TO THOSE PERSONS INVOLVED IN THE TRIAL WHO HAVE A NEED TO KNOW, WITH THE OBLIGATION NOT TO FURTHER DISSEMINATE THIS INFORMATION. THESE RESTRICTIONS ON DISCLOSURE WILL APPLY EQUALLY TO ALL FUTURE ORAL OR WRITTEN INFORMATION SUPPLIED TO YOU BY THE SPONSOR OR ITS AFFILIATES OR REPRESENTATIVES THAT IS DESIGNATED AS “PRIVILEGED” OR “CONFIDENTIAL”.

2. SYNOPSIS

TITLE OF TRIAL: Examination of the effect of ezetimibe on glucose metabolism
- Randomized, double-blind, placebo-controlled study in type 2 diabetes mellitus patients with hypercholesterolemia - Phase4, Protocol No.367(also known as SCH 58235, P06541)

OBJECTIVES:

Primary Trial Objective:
The primary objective is to examine the safety of ezetimibe, compared to placebo, on glucose metabolism with regard to change in HbA_{1c} from baseline to the end of treatment in patients with type 2 diabetes and hypercholesterolemia.

Supportive Safety Objectives for the Primary: To compare ezetimibe to placebo for:

(1) change in glycoalbumin and fasting plasma glucose from baseline to the end of treatment.

(2) proportion of patients having onset of the adverse event "exacerbation of diabetes mellitus" (exacerbation of diabetes mellitus will be judged carefully taking into account the index of blood glucose control, diabetes medications, and compliance to diet and exercise therapy)

(3) proportion of patients with changes to diabetes medications due to worsening of diabetes (However, small changes in insulin dosing +/-5U are excluded)

Supportive Efficacy Objectives:

To examine percent change (%) of ezetimibe compared to placebo in serum lipids (LDL-cholesterol, total cholesterol, triglycerides, HDL-cholesterol, and non–HDL-cholesterol) from baseline to the end of treatment.

Trial Design

Overview:
This is a randomized, double-blind, placebo-controlled, parallel-group, multi-site trial of ezetimibe in patients with type 2 diabetes and hypercholesterolemia to be conducted in conformance with Good Clinical Practices.

Number of Trial Centers: Approximately 20

Duration of Participation:
Each subject will participate in the trial for approximately 33 weeks from the time the subject signs the Informed Consent Form (ICF) through the final contact. After a screening phase of 5 weeks each subject will be receiving assigned treatment for approximately 24 weeks. Serious adverse events should be monitored from the time informed consent is obtained to 30 days after the administration of the last dose of study drug.

Duration of Trial:
The trial will require approximately 18 months from the beginning to the end of the overall trial (first subject signing informed consent to last contact with last subject).

Key Inclusion/Exclusion Criteria:

Subjects with a diagnosis of type 2 diabetes mellitus and hypercholesterolemia will be selected to participate in the trial.

Key Inclusion Criteria:

(1) Patients who have been diagnosed as having type 2 diabetes mellitus and undergoing treatment with oral anti-diabetic drugs or insulin or both.

(2) Patients who had no change in the type, dose, and regimen of drugs for the treatment of diabetes within 12 weeks before Visit 1 (the start of the screening phase). However, small changes in insulin dosing +/-5U are acceptable.

(3) Patients who have been undergoing diet and exercise therapy with no change in either therapy within 4 weeks before Visit 1 (however, exercise therapy will not necessarily be applicable in the case of patients with coexisting conditions judged not appropriate to meet this criterion).

(4) Patients whose LDL-cholesterol level measured at Visit 1 (the start of the screening phase) is less than 140 mg/dl in the case of patients who had been receiving serum lipid lowering drugs before the start of the screening phase and less than 160 mg/dl in the case of patients who had not been receiving such drugs

(5) Patients whose LDL-cholesterol level measured at Visit 2 is 120 mg/dl or more and less than 160 mg/dl.

Key Exclusion Criteria:

(1) Patients with triglyceride value exceeding 400 mg/dl at Visit 1 or Visit 2.

(2) Patients with HbA_{1c} value of 8.4% or more at Visit 1 or Visit 2. (HbA_{1c} will be recorded in NGSP.)

TITLE OF TRIAL: Examination of the effect of ezetimibe on glucose metabolism
- Randomized, double-blind, placebo-controlled study in type 2 diabetes mellitus patients with hypercholesterolemia - Phase4, Protocol No.367(also known as SCH 58235, P06541)

(3) Patients with fasting plasma glucose of 170 mg/dl or more at Visit 1 or Visit 2.

INVESTIGATIONAL PRODUCT, DOSE, MODE OF ADMINISTRATION

Investigational Product: Ezetimibe 10 mg will be administered orally once daily for 24 weeks from the start of the treatment phase.

Reference Product: Placebo will be administered orally once daily for 24 weeks from the start of the treatment phase.

STATISTICAL METHODS:

Data Sets to be Analyzed:

(1) Data Set for Safety

The data set for the statistical analysis of safety is the Per Protocol Set (PPS). Missing values will not be replaced with other values. The Full Analysis Set (FAS) will be used for sensitivity analysis of the primary trial objective.

1) Full Analysis Set (FAS): FAS is defined as all randomized subjects excluding the following:
- Subjects from whom written informed consent for participation in this study was not obtained
- Subjects who have not been diagnosed with type 2 diabetes mellitus
- Subjects who are not undergoing treatment with oral diabetes drugs or insulin
- Subjects who did not receive any study drug during the study period
- Subjects for whom no baseline data (Visit 2) were obtained or if obtained, no subsequent data were obtained

2) Per Protocol Set (PPS): PPS is defined as all randomized subjects excluding the following (also, data of HbA_{1c}, glycoalbumin and fasting plasma glucose after change in diabetes medication will not be used):
- Subjects excluded from FAS
- Subjects in violation of inclusion or exclusion criteria
- Subjects with a medication rate (days study drug was taken/stipulated days of study drug administration) of less than 75%

3) All subjects treated (AST): Since the analysis of adverse events and adverse drug reactions should be as comprehensive as possible, all subjects who take at least one dose of the study drug during the treatment period will be included in the population for analysis.

(2) Data Set for Efficacy

The data set for the statistical analysis of efficacy is FAS. Missing values will not be replaced with other values.

Sample Size:

Number of patients: 148 (at least 45 patients, accounting for at least 30% of the 148 patients, will be patients receiving insulin)

[Justification]

The difference between the ezetimibe and placebo groups in least squares mean of the amount of change in HbA_{1c} from baseline (Visit 2) to 24 weeks after treatment is set at 0.1%, with standard deviation of 0.7% and non-inferiority margin of 0.5%. With this hypothesis, the number of patients per group is 66 (total: 132 patients) with two-sided 5% (one-sided 2.5%) probability of type 1 error, 90% power, and 1:1 allocation to the ezetimibe and placebo groups. The sample size was set at a total of 148 patients to take dropouts (10%) into account.

Efficacy Analysis:

For LDL-cholesterol, total cholesterol, triglycerides, HDL-cholesterol, and non-HDL-cholesterol, the percent change from baseline at each measurement time point and summary statistics of measured values will be calculated by treatment group. For the percent change, analysis will be conducted using the same model of longitudinal analysis of covariance used for the primary safety endpoint.

Safety Analysis:

(1) Method of analysis of the primary endpoint and supportive endpoints

TITLE OF TRIAL: Examination of the effect of ezetimibe on glucose metabolism
- Randomized, double-blind, placebo-controlled study in type 2 diabetes mellitus patients with hypercholesterolemia - Phase4, Protocol No.367(also known as SCH 58235, P06541)

1) Primary endpoint (HbA_{1c}) and supportive endpoints of glycoalbumin and fasting plasma glucose

The longitudinal analysis of covariance will be used. In this model, baseline, treatment group, HbA_{1c} (2 categories: less than 7.4%, 7.4% to less than 8.4%), insulin use (2 categories: yes, no), time, "time x treatment group" interaction will be included. So as not to place restriction on the time-course curve of mean values, time will be a categorical variable. Between-group difference in mean amount of change from baseline at measurement time points will be estimated and tested based on this model. Correlation between time-course measured values will be modeled using unstructured covariance matrix, but if calculation does not converge, Toeplitz will be used for correlation.

Based on the above model, the difference in least squares mean from the placebo group and standard error as well as the 95% confidence interval will be calculated. The least squares mean value of the amount of change from baseline with each group and standard error as well as the 95% confidence interval will also be calculated. As sensitivity analysis of the primary endpoint, the same analysis will be conducted with FAS.

2) Other supportive endpoints

Between-group comparison will be conducted using Fisher's exact test for the proportion of subjects having onset of "exacerbation of diabetes" as an adverse event. Between-group comparison using Fisher's exact test will also be conducted for the proportion of subjects with changes to diabetes medications due to worsening of diabetes.

(2) Other analytical methods

1) Summary statistics

Summary statistics are the number of patients, mean value, standard error, median value, minimum value, and maximum value. Standard deviation will be calculated in place of standard error for demographic variables and baseline characteristics.

2) Adverse events and adverse drug reactions

The number of subjects with adverse events and the incidence of adverse events will be presented by treatment group. The number and incidence of adverse events by system organ class and event (symptoms and findings) will also be tabulated. For each event, tabulation by severity classification (mild, moderate, severe, life-threatening) will also be conducted. Tabulation will also be conducted of the number and incidence of serious adverse events and significant adverse events.

The same tabulations will be conducted for adverse drug reactions.

If the same symptom occurs in multiple degrees of severity in a single patient, tabulation will be conducted based on the most severe degree of severity.

3) Laboratory test values, vital signs, etc.

1. Tabulation of metric data

For the laboratory test values and measurement values below, summary statistics will be calculated by treatment group and by measurement time point (before treatment to after 24 weeks of treatment). A line graph presenting time-course change of individual patients from before treatment to after 24 weeks of treatment will also be prepared.

- Glucose metabolism (HbA_{1c}, glycoalbumin, fasting plasma glucose)
- Hematology (WBC count, RBC count, hemoglobin, hematocrit, platelet count)
- Biochemistry (AST, ALT, γ-GTP, alkaline phosphatase, LDH, CPK, total bilirubin, direct bilirubin, total protein, BUN, uric acid, creatinine, Na, K, Cl)
- Vital signs (body weight, blood pressure, pulse)

2. Judgment of normal/abnormal

Incidence will be tabulated by treatment group and by measurement time point.

Interim Analysis: No formal interim analyses are planned.

2.1 Trial Design Diagram

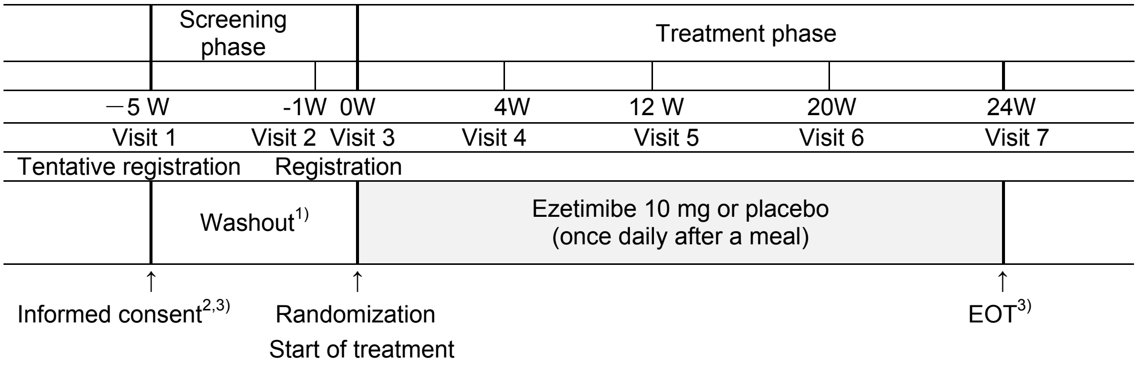

1): Washout of lipid lowering drugs
2): At Visit 1 or before
3): Serious adverse events should be monitored from the time informed consent is obtained to 30 days after the administration of the last dose of study drug.

2.2 Trial Flow Chart

Check and test Item			Screening phase			Treatment phase			
			Tentative registration	Test	Registration	Administration of study medication			
Visits			-5 W[1)]	-1 W[2)]	0 W	4 W[3)]	12 W[3)]	20W[3)]	24 W[4)]
Visit Number			1	2	3	4	5	6	7
Patient background			←	O	→				
Oral inquiry	Compliance to medication schedule					O	O	O	O
Adverse events*						O	O	O	O
Concomitant drugs, drugs for treatment of diabetes			O		O	O	O	O	O
Height[5)], Body weight, blood pressure, pulse			O		O	O	O	O	O
Laboratory tests	Glucose metabolism	HbA_{1c}	O	O		O	O	O	O
Laboratory tests	Glucose metabolism	Glycoalbumin, fasting plasma glucose	O	O		O	O	O	O
Laboratory tests	Serum lipids	LDL-C, TC, TG, HDL-C, non-HDL-C	O	O		O	O	O	O
Laboratory tests	Hematology	WBC, RBC, hemoglobin, hematocrit, platelets	O	O			O		O
Laboratory tests	Chemistry	AST, ALT, γ-GTP, alkaline phosphatase, LDH, CPK, total bilirubin, direct bilirubin, total protein, BUN, creatinine, uric acid, Na, K, Cl	O	O		O	O	O	O
Laboratory tests	Endocrinology	TSH	O						
Laboratory tests	Pregnancy test	Urine hCG**	O	O		O	O	O	O
Dispense Study Drug					O	O	O	O	

*: Serious adverse events should be monitored from the time informed consent is obtained to 30 days after the administration of the last dose of study drug.

**: Measured only in female subjects for whom pregnancy is possible. Urine hCG will be determined locally, serum hCG test is determined by central lab if urine pregnancy performed at the site is positive.

1): Allowance for a different day of visit is -2 weeks at 5 weeks before the start of the treatment phase.

2): Visit 2 will be made during the period from 5 days to 9 days before the start of administration if the study drug.

3): Allowance for a different day of visit is ±1 week at 4, 12 or 20 weeks.

4): Allowance for a visit is +2 weeks at 24 weeks after the start of the treatment period.

5): Height will be measured at visit 1.

Check and test items stipulated for Week 24 will be conducted at discontinuation.

3. TABLE OF CONTENTS

1. TITLE PAGE 1
2. SYNOPSIS 3
2.1 Trial Design Diagram 6
2.2 Trial Flow Chart 7
3. TABLE OF CONTENTS 8
3.1 List of Appendices 11
4. LIST OF ABBREVIATIONS AND DEFINITIONS OF TERMS 12
5. INTRODUCTION 14
5.1 Therapeutic Rationale 14
5.2 Subject Population Rationale 15
5.3 Dose and Administration Rationale 16
6. TRIAL OBJECTIVES 16
6.1 Primary Trial Objective 16
6.2 Supportive Safety Objectives for the Primary 17
6.3 Supportive Efficacy Objectives 17
7. INVESTIGATIONAL AND ANALYSIS PLAN 17
7.1 Overall Trial Design 17
7.2 Beginning and End of the Trial 18
7.3 Trial Population 19
7.3.1 Subject Inclusion Criteria 19
7.3.2 Subject Exclusion Criteria 22
7.3.3 Subject Discontinuation Criteria 23
7.3.4 Replacement of Subjects 26
7.4 Treatments 26
7.4.1 Trial Treatments 26
7.4.2 Non-Trial Treatments 31
7.4.3 Procedures for Monitoring Subject Compliance With Administration of Trial Treatments 33
7.5 Trial Schedule 33
7.6 Trial Procedures 34
7.7 Assessments 37
7.7.1 Efficacy Assessments 37
7.7.2 Safety Monitoring and Assessments 38
7.8 Criteria for Early Termination of the Trial 46
8. STATISTICAL AND ANALYTICAL PLAN 46
8.1 Person-in-charge of Statistical Analysis and In-house Blinding 46
8.2 Items of Analysis 46

8.2.1 Items of Safety Analysis ... 46
8.2.2 Items of Efficacy Analysis ... 47
8.2.3 Derivations of Safety Endpoints ... 48
8.3 Data Sets ... 48
8.3.1 Data Set for Safety ... 48
8.3.2 Data Set for Efficacy ... 49
8.4 Statistical Procedures ... 49
8.4.1 Statistical Methods for Safety ... 49
8.4.2 Statistical Methods for Efficacy ... 52
8.4.3 Other analyses ... 52
8.5 Multiplicity ... 53
8.6 Sample size determination and power ... 53
8.7 Sub-group Analysis ... 53
8.8 Interim Analysis ... 54
8.9 Handling of Patients ... 54
9. ADHERENCE TO ETHICAL, REGULATORY, AND ADMINISTRATIVE CONSIDERATIONS ... 54
9.1 Ethical Conduct of the Trial ... 54
9.1.1 Independent Ethics Committee or Institutional Review Board ... 54
9.1.2 Subject Information and Consent ... 55
9.1.3 Subject Identification Card ... 55
9.1.4 Registration of the Trial ... 56
9.2 Reporting Trial Data to the Sponsor ... 56
9.2.1 Data Collection Forms ... 56
9.2.2 Preparing Case Report Forms for All Subjects ... 57
9.2.3 Preparing Case Report Forms for Subjects Who Fail Screening ... 57
9.3 Publications and Other Rights ... 58
9.3.1 Rights to Publish by the Investigator ... 58
9.3.2 Use of Proprietary or Confidential Information in a Publication ... 58
9.3.3 Use of Trial Information in a Publication ... 59
9.3.4 Authorship of Publications ... 59
9.4 Trial Documents and Records Retention ... 60
10. INVESTIGATORS AND TRIAL ADMINISTRATIVE STRUCTURE ... 61
10.1 Sponsor ... 61
10.2 Investigators ... 61
10.2.1 Selecting Investigators ... 61
10.2.2 Financial Disclosure Requirement ... 61
10.2.3 Clinical Study Report Coordinator Investigator ... 61

10.3 Central Organizations 62
11. REFERENCES 62

3.1 List of Appendices

Appendix 1 Code of Conduct for Clinical Trials 64

4. LIST OF ABBREVIATIONS AND DEFINITIONS OF TERMS

Term	Definition
AE	Adverse Event
ALP	Alkaline Phosphatase
ALT	Alanine aminotransferase (SGPT)
AST	Aspartate aminotransferase (SGOT)
BMI	Body Mass Index
CFR	Code of Federal Regulations
Cl	Chlorine
CPK	Creatine Phosphokinase
CRF	Case Report Form
CSGS	Clinical Schedule Generation System – Merck interactive, computerized system for the generation and retention of allocaton and component schedules used in clinical trials, for all phases.
CSR	Clinical Study Report
CTD	Clinical Trial Directive
EU	European Union
FAS	Full Analysis Set
FDA	Food and Drug Administration, USA
Safety Data Reporting Form 1727	The sponsor's collection form used to report serious adverse events (SAE) or other events to Global Safety in the event that the EDC system is not functioning or able to accept SAE reports. The form is accompanied by the Safety Data Reporting Form 1727 Completion Guide and Instructions. SAE information can also be provided by means of a suitable alternative as long as it contains the equivalent information and is approved by Global Safety.
GCP	Good Clinical Practice
γ-GTP	gamma-glutamyl transpeptidase
HbA_{1c}	Hemoglobin A_{1c}
hCG	Human Chorionic Gonadotropin
ICH	International Conference on Harmonisation of Technical Requirements for Registration of Pharmaceuticals for Human Use
IEC	Independent Ethics Committee
ICMJE	International Committee of Medical Journal Editors
IMP	Investigational Medicinal Product
IND	Investigational New Drug Application; legal instrument in the USA that allows trial of unapproved, investigational new drugs in human subjects
Investigational Product	The drug, biologic, and/or device being investigated in the current trial
K	Potassium
IRB	Institutional Review Board

Term	Definition
LDH	Lactate Dehydrogenase
Na	Sodium
PPS	Per Protocol Set
RBC	Red Blood Cell
RSI	Reference Safety Information
TSH	Thyroid Stimulating Hormone
SAE	Serious Adverse Event
(S)AE	All Adverse Events, including Serious Adverse Events
SGOT	Serum Glutamic Oxaloacetic Transaminase (AST)
SGPT	Serum Glutamic Pyruvic Transaminase (ALT)
SOP	Standard Operating Procedure
USA	United States of America
WBC	White Blood Cell

5. INTRODUCTION

Redacted

Redacted

Redacted

6. TRIAL OBJECTIVES

6.1 Primary Trial Objective

The primary objective is to examine the safety of ezetimibe, compared to placebo, on glucose metabolism with regard to change in HbA_{1c} from baseline to the end of treatment in patients with type 2 diabetes and hypercholesterolemia.

Primary Non-inferiority Hypothesis:

For the primary safety hypothesis, the ezetimibe treatment group will be considered non-inferior to the placebo control group if the upper bound of the two-sided 95% confidence interval (CI) of the between-treatment difference (ezetimibe minus placebo) in means for change in HbA_{1c} from baseline to the end of treatment does not exceed 0.5%.

6.2 Supportive Safety Objectives for the Primary

The objectives are to compare ezetimibe to placebo for:

(1) change in glycoalbumin and fasting plasma glucose from baseline to the end of treatment.

(2) proportion of patients having onset of the adverse event “exacerbation of diabetes mellitus” (exacerbation of diabetes mellitus will be judged carefully taking into account the index of blood glucose control, diabetes medications, and compliance to diet and exercise therapy)

(3) proportion of patients with changes to diabetes medications due to worsening of diabetes (However, small changes in insulin dosing +/-5U are excluded)

6.3 Supportive Efficacy Objectives

The objectives are to examine percent change (%) of ezetimibe compared to placebo in serum lipids (LDL-cholesterol, total cholesterol, triglycerides, HDL-cholesterol, and non–HDL-cholesterol) from baseline to the end of treatment.

7. INVESTIGATIONAL AND ANALYSIS PLAN

7.1 Overall Trial Design

This is a randomized, double-blind, placebo-controlled, parallel-group, multi-site trial of ezetimibe in patients with type 2 diabetes and hypercholesterolemia.

The trial design is appropriate for the indication studied. Validated methods of data collection, analysis, and evaluation will be used for the trial.

7.2 Beginning and End of the Trial

Each subject is considered to be enrolled in the trial when the subject (or the subject’s legal representative) has provided written informed consent.

Each subject is considered to have ended participation in the trial when he/she has completed the last protocol-specified contact (eg, visits or telephone contacts) or prematurely discontinues from the study.

A subject is considered to have completed the trial if the subject has completed all of the protocol-specified visits and activities.

A subject is considered to have discontinued after he/she has withdrawn consent or has been discontinued under the conditions specified in **Section 7.3.3**.

A subject is considered to have been lost to follow-up if he/she is unable to be contacted by the investigator. The end of participation for a subject lost to follow-up is the last known contact (eg, visit or telephone contact).

The overall trial begins when the first subject is enrolled (ie, signs the informed consent form). The overall trial ends when the last remaining subject has ended participation in the trial, by completing the trial, being discontinued from the trial, or being lost to follow-up.

Each subject will be monitored for the occurrence of SAEs immediately after the subject has signed informed consent. Each subject will be followed for serious adverse events for up to and including 30 days after the last dose. Follow-up procedures related to pregnancy or SAEs may continue beyond the end of the clinical trial.

Once a subject has ended participation in the trial, the investigational product(s) from the trial will no longer be available to the subject and any future care will be provided according to the subject's personal physician.

Each subject will participate in the trial for approximately 33 weeks from the time the subject signs the Informed Consent Form (ICF) through the final protocol-specified contact. After a screening phase of 5 weeks each subject will receive assigned treatment (**Section 7.4.1.1**) for approximately 24 weeks. Serious adverse events should be monitored from the time informed consent is obtained to 30 days after the administration of the last dose of study drug.

The duration of trial is described in a separately prepared accompanying sheet.

7.3 Trial Population

Subjects with a diagnosis of type 2 diabetes and hypercholesterolemia will be selected to participate in the trial.

7.3.1 Subject Inclusion Criteria

A subject must meet all the criteria listed below to participate in the trial. The judgment for laboratory test-related criteria will be based on the results of tests conducted by the central laboratory.

1. Hypercholesterolemia patients who have been diagnosed as having type 2 diabetes and undergoing treatment with oral anti-diabetic drugs or insulin or both.
2. Patients who had no change in the medication (drugs, dose and administration) for the treatment of diabetes within 12 weeks before Visit 1 (the start of the screening phase). However, small changes in insulin dosing +/-5U are acceptable.
3. Patients who have been undergoing diet and exercise therapy with no change in either therapy within 4 weeks before Visit 1 (however, exercise therapy will not necessarily be applicable in the case of patients with coexisting conditions judged not appropriate to meet this criterion).
4. Patients whose LDL-cholesterol level measured at Visit 1 (the start of the screening phase) is less than 140 mg/dl in the case of patients who had been receiving serum lipid lowering drugs before the start of the screening phase and less than 160 mg/dl in the case of patients who had not been receiving such drugs.
5. Patients whose LDL-cholesterol level measured at Visit 2 (7 days before the start of the treatment phase) is 120 mg/dl or more and less than 160 mg/dl.
6. Each subject must be ≥ 20 to ≤ 75 years of age (as of the day of obtaining informed consent).
7. Either sex.

   Each sexually active female subject of child-bearing potential must agree to use a medically accepted method of contraception from informed consent to the last dose of study drug.

   Medically accepted methods of contraception include condoms (male or female) with or without a spermicidal agent, diaphragm or cervical cap with spermicide, medically prescribed IUD, inert or copper-containing IUD, hormone-releasing IUD, systemic hormonal contraceptive, and surgical sterilization (eg, hysterectomy or tubal ligation).

A female subject who is not of reproductive potential is eligible without requiring the use of contraception. A female subject who is not of reproductive potential is defined as: one who has either 1) reached natural menopause defined as age 46 or older with 12 months of spontaneous amenorrhea, 2) 6 weeks post surgical bilateral oophorectomy or hysterectomy, or 3) bilateral tubal ligation.

8. Outpatients
9. Each subject must be able to adhere to dose and visit schedules.
10. Each subject must be willing and able to provide written informed consent for the trial.

[Rationale]

(1) Hypercholesterolemia patients with type 2 diabetes were selected based on the objective of this study. Fasting plasma glucose and HbA_{1c} are known to be indices of diabetes, but no specific values are cited as selection criteria since, depending on treatment conditions, there are cases when normal values are observed.

(2)-(3) These were set to select subjects with stable diabetes since the objective of this study is to evaluate effect on glucose metabolism.

(4)-(5) The lipid management goal for Japanese patients with hypercholesterolemia is presented in the “Guideline for the Prevention of Atherosclerotic Diseases (2007)”. Since this study is in patients with coexisting diabetes mellitus who are high-risk patients, the LDL-cholesterol value measured at Visit 2 (7 days before the start of the treatment phase) was set to be at least 120 mg/dl, as the goal of LDL-cholesterol value in patients with coexisting diabetes mellitus is less than 120 mg/dl. An upper limit was also set, at less than 160 mg/dl measured at Visit 2, since one-half of the enrolled subjects will receive placebo for 24 weeks. This upper limit was set referring to the U.S. NCEP hypercholesterolemia criteria which indicate a 2-time higher relative risk for clinical cardiovascular events and death with total cholesterol of 240 mg/dl (LDL-cholesterol of 160 mg/dl) compared to total cholesterol of 200 mg/dl (LDL-cholesterol of 120 mg/dl) based on the MRFIT relationship between total cholesterol value and death rate due to coronary artery disease [8), 9)].

Inclusion criteria (4) was set to prevent washout of serum lipid lowering drugs of subjects with high LDL-cholesterol levels highly likely not to meet inclusion criteria (5) by judging the possibility of subjects meeting the upper limit of the inclusion criteria at the start of the observation period (Visit 1).

Lipid management goals

Patient category			Lipid management goal
		Main coronary risk factors other than LDL-c[a]	LDL-cholesterol value
Primary prevention[b]	I (low risk)	0	<160
	II (mid-risk)	1 or 2	<140
	III (high risk)	3 or more	<120
Secondary prevention[c]	History of coronary artery disease		<100

a: Main coronary risk factors other than LDL-cholesterol: (1) age (males ≥45, females ≥55), (2) family history of coronary artery disease, (3) smoking, (4) hypertension, (5) diabetes (including glucose tolerance abnormality), (6) low HDL-cholesterol levels (<40 mg/dl)

b: Drug treatment will be considered after first instituting improvement of life-style.

c: Drug treatment will be considered together with lifestyle improvement.

Note: Category III if there is coexisting diabetes, cerebral infarction, or arteriosclerosis obliterans.

(From Guideline for Prevention of Atherosclerotic Diseases 2007)[2)]

(6) The upper age limit was set at 75 years since there is a possibility that the evaluation of the safety on glucose metabolism may not be accurate if old patients are included.

(7) No gender-dependent difference in efficacy or safety data was observed in the results of Japanese and non-Japanese clinical studies.

(8) Subjects to participate in this study do not have serious coexisting conditions.

(9)-(10) These were set to conduct this trial to comply with GCP and protocol.

7.3.2 Subject Exclusion Criteria

A subject meeting any of the exclusion criteria listed below must be excluded from participating in the trial. The judgment for laboratory test-related criteria will be based on the results of tests conducted by the central laboratory:

1. Patients with triglyceride value exceeding 400 mg/dl at Visit 1 or Visit 2.
2. Patients with HbA_{1c} value of 8.4% or more at Visit 1 or Visit 2. (HbA_{1c} will be recorded in NGSP.)
3. Patients with fasting plasma glucose of 170 mg/dl or more at Visit 1 or Visit 2.
4. Patients with a coexisting disease (hemoglobinopathy, hemolytic anemia, etc.) which may affect HbA_{1c} measurement.
5. Patients with CPK value >2 X ULN at Visit 1 or Visit 2.
6. Patients with active liver disease, or ALT and/or AST>2 X ULN at Visit 1 or Visit 2.
7. Patients who have received the lipid lowering drugs during the screening phase or have change in the medication (drugs, dose and administration) for the treatment of diabetes during the screening phase.
8. Patients who donated blood within 4 weeks before Visit 1.
9. Patients who plan to undergo elective surgery.
10. Patients with homozygous or heterozygous hypercholesterolemia.
11. Patients with or with a history of coronary artery disease, stroke (excluding lacunar infarct), or arteriosclerosis obliterans.
12. Patients who have previously received ezetimibe.
13. Patients with a history of hypersensitivity to investigational product or its excipients.
14. Patients with hypercholesterolemia associated with:
    - Hypothyroidism
    - Obstructive gall bladder or biliary disease
    - Chronic renal failure
    - Pancreatitis
15. Patients with a history of cancer within the past 5 years from Visit 1 (except for successfully treated dermatological basal cell or squamous cell carcinoma of in situ cervical cancer).
16. Patients with hyperlipidemia caused by drugs having adverse effect on serum lipids.
17. Patients who are using cyclosporine or systemic corticosteroids within 4 weeks before Visit 1.

18. Patients who are currently participating in another interventional clinical trial or have received an investigational drug within 4 weeks before Visit 1.
19. Patients who are nursing, pregnant, or intending to become pregnant.
20. The subject or a family member is among the personnel of the investigational or sponsor staff directly involved with this trial.
21. Patient has any condition or situation which, in the opinion of the investigator (sub-investigator), might pose a risk to the patient or interfere with participation in the study.

[Rationale]

(1) If triglyceride value exceeds 400 mg/dl, it may not be possible to accurately measure serum lipids, etc. due to serum becoming cloudy.

(2),(3) This is set to exclude patients with high possibility of having diabetes which is not well-controlled since the objective of this study is to evaluate effect on glucose metabolism.

(4) This is set to exclude patients having coexisting conditions which may have an effect on HbA_{1c} measurement values.

(5)-(18) These were set to evaluate the efficacy and safety of the test product correctly.

(19) This is set since safety of administration in pregnancy and to the fetus and neonates has not been established.

(20) This was set to ensure the objectivity of the study.

(21) This was set to assure the safety of subjects and to exclude subjects with whom it is considered difficult for the investigator (sub-investigator) to perform the study in compliance with the study protocol.

7.3.3 Subject Discontinuation Criteria

A subject may discontinue from the clinical trial at any time for any reason.

It is the right and the duty of the investigator or subinvestigator to stop treatment in any case in which emerging effects are of unacceptable risk to the individual subject. In addition, the investigator or subinvestigator is to stop treatment of any subject with unmanageable factors that may interfere significantly with the trial procedures and/or the interpretation of results.

Discontinuation is permanent : once a subject is discontinued, he/she shall not be allowed to enroll again.

At a minimum collect the following information when a subject discontinues:

1. The reason the subject discontinued;
2. The date of the last dose of test products from the trial;
3. The date of the last assessment and/or contact. A follow-up contact (telephone or visit) will be arranged as appropriate;
4. (Serious) Adverse events;
5. Compliance with the test product administration as specified in this protocol;
6. Final Assessments:
   Every effort should be made to ensure that all procedures and evaluations scheduled for the final trial visit are performed (**Section 2.2**, Trial Flow Chart)
7. Retrieve all investigative products and test articles from the subject.

A subject must be discontinued from the trial for any of the following reasons:

(1) Discontinuation criteria from obtaining informed consent to the start of the treatment phase
In the case of discontinuation after informed consent is obtained for the following reasons, the investigator (sub-investigator) will prepare the CRF and record the reason for discontinuation in the CRF and in the remarks column of the subject screening log.

1) LDL-cholesterol measured at Visit 1 (the start of the screening phase) is 140 mg/dl or more in the case of patients who had been receiving serum lipid lowering drugs before the start of the screening phase and 160 mg/dl or more in the case of patients who had not been receiving such drugs.

2) LDL-cholesterol measured at Visit 2 (7 days before the start of the treatment phase) is less than 120 mg/dL or 160 mg/dL or more.

3) If any of the laboratory test values below measured at Visit 1 or Visit 2 is applicable:

Laboratory test items	Criteria
Triglycerides	> 400 mg/dL
AST	> 2x ULN
ALT	> 2x ULN
CPK	> 2x ULN
HbA_{1c}	≥8.4%
FPG	≥170 mg/dL

4) It is found that the subject does not meet all of the inclusion criteria or if any of the exclusion criteria is found applicable.

5) The subject or legal representative (such as a parent or legal guardian) withdraws consent.

6) The subject changes his/her residence, changes to another hospital, or does not visit the hospital.

7) It is judged that starting treatment is undesirable due to deterioration of the underlying disease, an unforeseen accident, or incidental symptoms, or the onset of adverse events (including serious or life-threatening adverse event).

8) It is judged that starting treatment is undesirable for other reasons.

(2) Discontinuation criteria after the start of the treatment phase

If any of the situations indicated below becomes applicable, the investigator (sub-investigator) will immediately contact the relevant subject to discontinue administration of the study drug. In addition, the investigator (sub-investigator) will instruct the subject to visit the hospital as early as possible, instructing them to bring the remaining study drugs when they make the hospital visit. The investigator (sub-investigator) will perform the observations, clinical laboratory tests, etc. to be conducted at Week 24 when the subject visits the hospital.

1. AST or ALT is >3 times the upper limit of the reference values on 2 consecutive occasions.

2. CPK exceeds 10 times the upper limit of the reference value.

3. It is found that the subject does not meet all of the inclusion criteria or if any of the exclusion criteria is found applicable.

4. The subject or legal representative (such as a parent or legal guardian) withdraws consent;

5. The subject changes his/her residence, changes to another hospital, or does not visit the hospital.

6. It is judged undesirable to continue administration because of deterioration of the underlying disease, an unforeseen accident, or incidental symptoms, or the onset of adverse events (including serious or life-threatening adverse event).

7. It is judged necessary to discontinue the study for other reasons.

7.3.4 Replacement of Subjects

A subject who discontinues from the trial will not be replaced.

7.4 Treatments

7.4.1 Trial Treatments

7.4.1.1 Treatments Administered

Each subject will receive trial treatment at Registration Day (Visit 3: start of the treatment phase) and is to take the first dose in the evening of that same day. Study Drug (Ezetimibe 10 mg or Placebo) will be administered orally after meal once daily for 24 weeks from the start of the treatment phase (Visit 3).

7.4.1.2 Method of Treatment Assignment, Randomization, and/or Stratification

Randomization of the study drugs will be conducted by CSGS. The method of assigning drug numbers to patients will be stipulated in a separate procedure (standard operating procedures of the patient registration center)

Patients will be assigned 1:1 to receive ezetimibe or placebo.

Randomized treatment assignment will be stratified for the following factors:

1. Two categories based on HbA_{1c} at Visit 2 (7 days before the start of the treatment phase) (less than 7.4% and 7.4% to less than 8.4%)
2. Two categories of “insulin” and “non-insulin”

7.4.1.3 Selection and Timing of Dose for Each Subject

7.4.1.3.1 Selecting the Dose for Each Subject

The rationale for the selection of doses to be used in this trial is presented in **Section 5.3**.

7.4.1.3.2 Determining the Timing of Dose Administration for Each Subject

The investigator (sub-investigator) will instruct patients on the administration method as follows:

(1) To be taken once daily after a meal. Whether it will be taken after breakfast, lunch, or dinner will be decided by the investigator or sub-investigator at the start of the treatment phase, and it will remain constant throughout the treatment phase.

(2) If the patient forgets to take the study drug after the predetermined meal, it will be taken after any meal during that day.

(3) Only on days of clinic visits, if the patient is to take the study drug after breakfast, administration will be after the next meal after the tests/observations conducted on the day of the visit.

(4) On days of clinic visits, no meals or beverages other than water should be ingested for 10 hours before the time of the visit.

(5) No infusion solution should be administered within 10 hours before the clinic visit (excluding infusion solution less than 50 ml and not containing sugar).

7.4.1.4 Blinding Trial Treatments

Ezetimibe and its matching placebo will be identical in appearance and will be packaged identically so that treatment blind is maintained. The placebo tablet is similar to the investigational product as regards appearance, weight, smell and taste. Neither the subject nor the sponsor and investigator (sub-investigator) will know which treatment the subject is receiving.

See **Section 7.7.2.6.4** for a description of the method of unblinding a subject during the trial, should such action be warranted.

7.4.1.5 Investigational Medicinal Products

The investigator shall take responsibility for and shall take all steps to maintain appropriate records and ensure appropriate supply, handling, storage, distribution, and usage of these materials in accordance with the protocol and any applicable laws and regulations.

7.4.1.5.1 Identity of Investigational Medicinal Product

(1) Name

Non-proprietary name: Ezetimibe

Chemical name:

(3R,4S)-1-(4-Fluorophenyl)-3-[(3S)-3-(4-fluorophenyl)-3-hydroxypropyl]-4-(4-hydroxyphenyl)azetidin-2-one

(2) Contents and drug form

Study drug	Contents	drug form
Investigational Product:	Each tablet contains 10 mg of ezetimibe	White-colored capsule-type tablet
Reference Product:	Each tablet does not contain ezetimibe	Placebo tablet of appearance identical to the investigational product.

(3) Composition

Study drug	Composition
Investigational Product:	It contains lactose hydrate, crystalline cellulose, povidone, croscarmellose sodium, sodium lauryl sulfate, and magnesium stearate, besides ezetimibe.
Reference Product:	It contains lactose hydrate, crystalline cellulose, povidone, croscarmellose sodium, sodium lauryl sulfate, and magnesium stearate.

(4) Storage method

The storage conditions will be indicated on the label.

The clinical supplies storage area at the site must be monitored by the site staff for temperature consistency with the acceptable storage temperature range as specified on the label or in the product label attached to the protocol. Documentation of temperature monitoring should be maintained.

7.4.1.5.2 Source

Investigational materials will be provided by the SPONSOR as summarized in the table below.

Product descriptions

Product Name & Potency	Dosage form
Ezetimibe (MK-0653) 10 mg or placebo	Tablet

7.4.1.5.3 Labeling

Supplies will be affixed with a clinical label in accordance with regulatory requirements.

7.4.1.5.4 Packaging

Each kit will contain 6 bottles of 35 tablets.

7.4.1.5.5 Storage

Trial treatment supplies must be stored in a secure, limited-access location under the storage conditions specified on the supply label. Site storage conditions should be monitored by the site personnel for adherence to label specifications and reviewed during site visits.

Documentation of temperature monitoring should be maintained.

7.4.1.5.6 Dispensing

The investigator or qualified designee(s) will dispense trial treatments at the designated site(s) to subjects who have provided written informed consent and have met the entry criteria. Clinical supplies may not be used for any purpose other than that which is stated in this protocol.

See the Trial Flow Chart in **Section 2.2** for a schedule of when clinical supplies are to be dispensed to the subjects.

7.4.1.5.7 Replacement of Investigational Product

Additional supplies will be delivered to sites as necessary..

7.4.1.5.8 Investigational Medicinal Product Accountability

Accurate and current accounting of the dispensing and return of investigational products will be maintained on an ongoing basis by a member of the trial site staff:

- Investigational medicinal products dispensed to each site will be recorded in the trial-specific Site Investigational Medicinal Product (IMP) Accountability Log (or equivalent document approved by the sponsor);
- Investigational medicinal products dispensed to each subject will be recorded in the trial-specific Subject IMP Accountability Log (or equivalent document approved by the sponsor).

The Site IMP Accountability Log and Subject IMP Accountability Log will be verified by the sponsor's trial monitor. The original Site IMP Accountability Log and Subject IMP Accountability Log will be approved by the investigator and retained at the trial site and a copy supplied to the sponsor when the trial is complete.

Each subject will be instructed by the investigator or designee to return all unused and partially used test articles to the site at all protocol-specified visits.

The sponsor’s trial monitor will instruct the site on the return of all investigational products supplies. A final inventory of the total amount of investigational products received at each trial site against the amount used and returned must be recorded in the Site IMP Accountability Log. Inventory records must be readily available for inspection by the trial monitor and/or auditor, and open to government inspection at any time.

7.4.2 Non-Trial Treatments

7.4.2.1 Prior and Concomitant Medications

7.4.2.1.1 Medications, Supplements, and Other Substances Prohibited Prior to Screening and During the Trial

Concomitant use of the following drugs is prohibited throughout the screening and treatment phases.

(1) The following hypolipidemic agents:

- HMG-CoA reductase inhibitor
- Anion-exchange resin
- Probucol
- Fibrates
- Nicotinic acid formulation
- EPA formulation
- Other serum lipid lowering agents

(2) Other study drugs

(3) Cyclosporin

(4) Systemic corticosteroids

[Rationale]

These were set to assure the safety of subjects and to allow the accurate evaluation of the safety and efficacy of the study drug.

The medications and other substances prohibited prior to screening are cited in Section 7.3.2.

7.4.2.1.2 Concomitant Medications, Supplements, and Other Substances Allowed During the Trial

(1) Concomitant Medications Allowed During the Study

Medications required to treat adverse events or concurrent illnesses are allowed during the study (except prohibited concomitant drugs).

Note that the use of any concomitant medication must relate to the documented medical history, prophylaxis, or an adverse event of the subject.

Drugs for the treatment of diabetes can be changed if the investigator or sub-investigator judges that a change is necessary based on the condition of the patient.

(2) Concomitant Therapies Allowed During the Study

Therapies required to treat adverse events or concurrent illnesses are allowed during the study.

Note that the use of any concomitant therapies must relate to the documented medical history, prophylaxis, or an adverse event of the subject.

1) Diet therapy

Diet therapy will be mandatory and there will be no change in the diet therapy during the study period. Patients will be instructed to continue with the diet therapy for diabetics they had been on at Visit 1 (the start of the screening phase).

2) Exercise therapy

Exercise therapy will be mandatory and there will be no change in the exercise therapy during the study period. Patients will be instructed to continue with the exercise therapy for diabetic they had been on at Visit 1. However, this will not necessarily be applicable in the case of patients with coexisting conditions or adverse events (arthritic disease of lower limbs, etc.) judged not appropriate to meet this criterion.

7.4.2.2 Other therapy

None.

7.4.3 Procedures for Monitoring Subject Compliance With Administration of Trial Treatments

At all protocol-specified visits, the investigator or qualified designee is to record whether treatment had been taken per protocol in the preceding interval. If not, the date(s) and reason for each dosing noncompliance must be recorded.

7.5 Trial Schedule

The visit-by-visit schedule of trial activities is provided in the Trial Flow Chart in **Section 2.2**.

The timing of each visit is relative to Day 1, which is defined as administration of the first dose of trial medication, Visit 3 (**Section 7.4.1.1**).

- Hospital visit 1 (start of the screening phase) will be made at 5 weeks before the start of the treatment phase. Allowance for a different day of visit is -2 weeks at 5 weeks before the start of the treatment phase.
- Hospital visit 2 (7 days before the start of the treatment phase) will be made during the period from 5 days to 9 days before the start of administration of the study drug (hospital visit 3).
- Hospital visit 3 (start of the treatment phase) is designated as Week 0, and is the baseline of the timing of each visit.
- Hospital visit 4-6 (4, 12, 20 weeks after the start of the treatment period) will be made at 4, 12, 20 weeks after the start of treatment period respectively. Allowance for a different day of visit is ±1 week at 4, 12 or 20 weeks.
- Hospital visit 7 (24 weeks after the start of the treatment period) will be made at 24 weeks after the start of treatment period respectively. Allowance for a visit is +2 weeks at 24 weeks after the start of the treatment period.
- After hospital visit 7 (after the end of the treatment period), physical examinations or additional examinations will be performed if the investigator (sub-investigator) judges it necessary due to the follow-up of adverse events, etc. The investigator (sub-investigator) judges if the additional examinations is performed in a fasted state or not.

All visits should be performed within the windows specified in **Section 2.2**, the Trial Flow Chart. Every attempt should be made to have each subject attend each visit as scheduled. However, if a subject is unable to attend a visit within the specified windows, the visit should be scheduled as closely as possible to these windows. A subject should not miss a protocol-specified visit due to scheduling difficulties.

7.6 Trial Procedures

The Trial Flow Chart in **Section 2.2** summarizes the trial procedures to be performed at each visit. Individual trial procedures are described below.

In order to minimize variability of evaluations, it is preferred that the same individuals perform the same types of evaluations for all subjects at each trial site.

If the condition of a patient or laboratory test values judged ineligible for inclusion in the study as a result of tests conducted at the start of the screening phase improves thereafter and it is possible that the patient is eligible for inclusion, informed consent will be newly obtained and tests to be conducted at the start of the screening phase will be allowed to be repeated once.

1. **Explain Trial and Obtain Written Informed Consent**

   The investigator or qualified designee will explain the trial to the subject, answer all of his/her questions, and obtain written informed consent before performing any trial-related procedure. A copy of the informed consent will be given to the subject (see Section 9.1.2 for further description of the Informed Consent).

   If it is necessary to discontinue a prohibited concomitant drug, it should be discontinued after written consent is obtained.

   The subject identification code will be assigned to each subject at the time of written informed consent.

2. **Issue or Collect Subject Identification Card**

   The investigator or qualified designee will provide the subject with a Subject Identification Card after the subject provides written informed consent. The investigator or qualified designee will retrieve the card from the subject at the last contact (see Section 9.1.3 for further description of the Subject Identification Card).

3. **Obtain Medical History**

A medical history will be obtained by the investigator or qualified designee. The following demographic factors will be investigated and recorded.

1) Year, month and date of birth
2) Sex
3) Race
4) Inpatient/outpatient
5) Menopause status (only for women)
6) Height, body weight
7) Duration of diabetes mellitus
8) History of treatment for diabetes (in the 12 weeks before the start of the screening phase [Visit 1])
9) History of treatment for hyperlipidemia (in the 4 weeks before the start of the screening phase)
10) Coexisting conditions (including transient diseases such as common cold during the observation period)
11) Experience of ezetimibe use
12) Presence/absence of a history of hypersensitivity to ingredients of ezetimibe tablets
13) Presence/absence of administration of other study drugs within 4 weeks before the start of the screening phase
14) Presence/absence of pregnancy or nursing
15) Presence/absence of the subject's wish to become pregnant

4. **Review Inclusion/Exclusion Criteria**

The inclusion and exclusion criteria will be reviewed by the investigator or qualified designee to ensure that the subject qualifies for the trial.

5. **Tentative Registration**

The investigator (sub-investigator) will conduct review based on the inclusion and exclusion criteria after obtaining informed consent. After confirming that the subject is eligible for inclusion in this study, the registration center will be contacted by fax to conduct tentative registration. The investigator (sub-investigator) and the sponsor will confirm that tentative registration has been completed by receipt by fax of "Notification of the result of tentative registration" from the registration center.

6. **Review Prior Medications**

Review of appropriate prior medications, including the necessary washout times, with the subject. A record of prior medication taken by the subject within 12 weeks before starting the trial is to be obtained.

7. **Registration**
   1) The investigator (sub-investigator) will conduct laboratory tests at Visit 2 (7 days before the start of the treatment phase).
   2) At Visit 3 (the start of the treatment phase), the investigator (sub-investigator) will confirm the results of laboratory tests of Visit 2. After confirming that the subject is eligible for inclusion in the study, registration will be conducted by fax.
   3) The registration center will assign the study drugs, and “Notification of registration”, providing the contents of the registration, patient number, and study drug number, will be sent by FAX to the investigator (sub-investigator) and sponsor.

8. **Record Concomitant Medications and Concomitant Therapies**

A record of medication taken by the subject during the trial is to be obtained.

(1) Concomitant Medications

Concomitant drugs used from the start of the screening phase to tests conducted at the end of the study or at discontinuation (up to 30 days after the end of administration if tests to be conducted at discontinuation occur more than 30 days after the end of administration) should be recorded in the case report form. Drugs used for the treatment of adverse events up to the day of outcome should be recorded.

(2) Concomitant Therapies

Concomitant therapies instituted from the start of the screening phase to tests conducted at the end of the study or at discontinuation (up to 30 days after the end of administration if tests to be conducted at discontinuation occur more than 30 days after the end of administration) should be recorded in the case report form. Therapies instituted for the treatment of adverse events up to the day of outcome should be recorded.

9. **Record (Serious) Adverse Events**

Adverse events occurring from the start of treatment to tests conducted at the end of the study or at discontinuation (up to 30 days after the end of administration if tests to be conducted at discontinuation occur more than 30 days after the end of administration) should be recorded in the case report form. Serious adverse events should also be recorded from the time informed consent is obtained to 30 days after the administration of the final dose of the study drug.

See **Section 7.7.2.4**, for instructions on the assessment and reporting of (Serious) Adverse Events and **Section 7.7.2.5** for instructions on the reporting of (Serious) Adverse Events to the sponsor.

10. **Height, Body Weight, Blood Pressure, and Pulse**

Body weight, blood pressure, and pulse will be measured and recorded.

Height will be measured and recorded at visit 1.

11. **Laboratory Tests**

Blood sampling will be conducted with patients in a fasted state. The following laboratory tests will be measured and recorded. The pregnancy test will only be conducted in female patients in whom pregnancy is possible. HbA_{1c} will be recorded in NGSP.

1) Glucose metabolism: HbA_{1c}, glycoalbumin, fasting plasma glucose
2) Serum lipids: LDL-cholesterol, total cholesterol, triglycerides, HDL-cholesterol, non-HDL-cholesterol
3) Hematology: WBC, RBC, hemoglobin, hemaocrit, platelets
4) Biochemistry: AST, ALT, γ-GTP, ALP, LDH, CPK, total bilirubin, direct bilirubin, total protein, BUN, creatinine, uric acid, Na, K, Cl
5) Endocrinology: TSH
6) Pregnancy test (urine): hCG

   Measured only in female subjects for whom pregnancy is possible. Urine hCG will be determined locally, serum hCG test is determined by central lab if urine pregnancy performed at the site is positive.

12. **Daily log form for recording drug administration**

The clinical study staff will provide the subject with a daily log form for recording drug administration and will direct the subject to keep a record of drug administration such as time of administration, number of tablets taken.

The subject must bring the daily log form and medication bottles (empty, partially used) to the hospital at each visit; and the clinical study staff or investigator/sub-investigator will check the status of the study drug administration.

7.7 Assessments

7.7.1 Efficacy Assessments

7.7.1.1 Supportive Efficacy Endpoint

Percent change (%) in LDL-cholesterol, total cholesterol, triglycerides, HDL-cholesterol, and non-HDL-cholesterol from baseline to the end of treatment

[Rationale]

The “Guidelines for the clinical evaluation method of antihyperlipidemic agents (1988)”[10)] indicates that using the percent change in lipids as an index is one method

of evaluation, and the percent change in LDL-cholesterol has been used as an evaluation method for the efficacy of serum lipid lowering agents in Japan and overseas. In addition, "Guidelines for Prevention of Atherosclerotic Diseases (2007)"[2)] indicates that the management of lipids is in principle evaluated by LDL-cholesterol values. Besides LDL-cholesterol, serum lipids are considered to be risk factors of arteriosclerosis, and the evaluation of the effect on serum lipids is considered to be appropriate as an index of efficacy.

7.7.2 Safety Monitoring and Assessments

7.7.2.1 Safety Endpoints

7.7.2.1.1 Primary safety Endpoint

The primary safety endpoint is related to the primary trial objective.

The primary safety endpoint is the amount of change in HbA_{1c} from baseline to the end of treatment.

If results are obtained indicating that the primary endpoint of HbA_{1c} does not increase with ezetimibe compared to placebo, it can be concluded that ezetimibe does not exacerbate diabetes even if increase in a supportive safety endpoints for the primary is observed with ezetimibe.

[Rationale]

In the Diabetes Treatment Guide [11)] (2010, Japan Diabetes Society [ed.], Bunkodo), the section on blood glucose control in the chapter on treatment goal and index of control indicates that:

"Importance is placed on HbA_{1c} as the index of blood glucose control and the primary evaluation should be conducted based on this index. HbA_{1c} is an index which reflects the average blood glucose values in the previous 1-2 months, and intra-individual variability is small. It is the most important index of blood glucose control."

It is therefore thought that HbA_{1c} is the most appropriate primary endpoint in this study.

7.7.2.1.2 Supportive Safety Endpoints for the Primary

- Change in glycoalbumin from baseline to the end of treatment

- Change in fasting plasma glucose from baseline to the end of treatment
- Proportion of patients having onset of the adverse event “exacerbation of diabetes mellitus” (exacerbation of diabetes mellitus will be judged carefully taking into account the index of blood glucose control, diabetes medications, and compliance to diet and exercise therapy)
- Proportion of patients with changes to diabetes medications due to worsening of diabetes (However, small changes in insulin dosing +/-5U are excluded)

7.7.2.1.3 Other Safety Endpoints

- Adverse events
- Adverse reactions
- Laboratory tests (hematology, chemistry)
- Vital signs (body weight, blood pressure, pulse)

7.7.2.2 Definition of Terms

7.7.2.2.1 Adverse Event

Per the International Conference on Harmonization (ICH), an adverse event (AE) is defined as any untoward medical occurrence in a patient or clinical investigation subject administered a pharmaceutical product and which does not necessarily have to have a causal relationship with this treatment. An AE can therefore be any unfavorable and unintended sign (including an abnormal laboratory finding, for example), symptom, or disease temporally associated with the use of a medicinal product, whether or not considered related to this medicinal product.

7.7.2.2.2 Adverse reaction

Adverse reactions are all undesirable or unintended reactions (including abnormal laboratory test values) to the study drug administered regardless of the dose. There must be some logical possibility of causal relationship between the adverse event and study drug administration, and they are reactions for which causal relationship cannot be ruled out.

7.7.2.2.3 Serious Adverse Event

Serious Adverse Event (SAE) is any untoward medical occurrence or effect that at any dose:

1. Results in death;
2. Is life-threatening;
3. Requires hospitalization or prolongation of existing inpatients' hospitalization;
4. Results in persistent or significant disability or incapacity; and/or
5. Is a congenital anomaly or birth defect;
6. Cancer.
7. Overdose（Whether accidental or intentional.）Any overdose whether or not associated with an adverse event must be reported.

Life-threatening in the definition of a serious adverse event refers to an event in which the subject was at risk of death at the time of event; it does not refer to an event which hypothetically might have caused death if it were more severe.

Medical judgment should be exercised in deciding whether an adverse event/reaction is serious in other situations. Important adverse events/ reactions that are not immediately life-threatening or do not result in death or hospitalization, but may jeopardize the subject or may require intervention to prevent one of the other outcomes listed in the definition above, should also be considered serious. These are considered "Other Important Medical Events".

7.7.2.2.4 Events of Clinical Interest

An "event of clinical intersest" is a non-serious adverse event or occurrence that is designated to be of special interest and must be reported to the sponsor as though it were a serious adverse event - as described in **Section 7.7.2.5.1**.

The following events are considered events of clinical interest for this trial:

An elevated AST or ALT lab value that is ≥3 x the upper limit of normal (ULN) and an elevated total bilirubin lab value that is ≥2 x ULN and, at the same time, an alkaline phosphatase lab value that <2 x ULN, as determined by way of protocol-specified laboratory testing or unscheduled laboratory testing.*

*Note: The guidance for assessment and follow up of these criteria can be found in the on a separately prepared guidance.

7.7.2.2.5 Overdose

An overdose is a significant variation above the recommended/scheduled dosage for a product. In this current trial an overdose of the investigational product ezetimibe is more than 1 tablet per day.

7.7.2.2.6 Clinical Supply Complaint

A Research and Commercialization Quality (RCQ) Clinical Complaint/GCP Inquiry Form is any written, electronic or oral communication that alleges a product defect. A Clinical Supply Complaint does not include Product Complaints alleging suspected product counterfeit, diversion, tampering or an Adverse Event.

7.7.2.2.7 Planned Hospitalization

A hospitalization planned by the subject prior to signing the ICF is considered a therapeutic intervention and not the result of a new SAE and should be recorded as medical history. If the planned hospitalization or procedure is executed as planned, the record in the subject's medical history is considered complete. However, if the event/condition worsens during the trial, it must be reported as an AE.

7.7.2.3 Monitoring

7.7.2.3.1 Monitoring Adverse Events

Each subject will be monitored for the occurrence of SAEs immediately after the subject has signed informed consent. Each subject will be followed for serious adverse events for up to and including 30 days after the last dose as described in **Section 7.2**.

Subjects will be questioned and/or examined by the investigator or a qualified designee for evidence of AEs. The questioning of subjects with regard to the possible occurrence of adverse events will be generalized such as, "How have you been feeling since your last visit?" The presence or absence of specific AEs should not be elicited from subjects.

Subjects having AEs will be monitored with relevant clinical assessments and laboratory tests, as determined by the investigator.

AEs, actions taken as a result of AEs, and follow-up results must be recorded in the electronic Case Report Forms (eCRF; **Section 9.2**), as well as in the subject's source documentation. Follow-up laboratory results should be filed with the subject's source documentation.

For all AEs that require the subject to be discontinued from the trial and SAEs, relevant clinical assessments and laboratory tests will be repeated as clinically appropriate, until final resolution or stabilization of the event(s).

7.7.2.3.2 Monitoring Laboratory Assessments

All laboratory assessments will be performed centrally at a certified laboratory selected by the sponsor. The clinical laboratory values will be reported to the investigator by the laboratory and he/she will review them for significance and consideration as an AE.

7.7.2.4 Assessment of Adverse Events

7.7.2.4.1 Assessment of Severity

Where the determination of adverse event severity rests on medical judgment, the determination of severity must be made with the appropriate involvement of a medically-qualified investigator.

The severity of AEs will be graded according to the following definitions:

Mild:	awareness of sign, symptom, or event, but easily tolerated;
Moderate:	discomfort enough to cause interference with usual activity and may warrant intervention;
Severe:	incapacitating with inability to do normal daily living activities or significantly affects clinical status, and warrants intervention;

7.7.2.4.2 Assessment of Causality

A medically-qualified investigator must assess the relationship of any AE (including SAEs) to the use of the investigational product using the guidelines listed below:

- Yes, there is reasonable possibility of drug relationship. There is evidence of exposure to test drug. The temporal sequence of the AE onset relative to the administration of the test drug is reasonable. The AE is more likely explained by the test drug than by another cause.
- No, there is not a reasonable possibility of drug relationship. Subject did not receive the test drug OR temporal sequence of the AE onset relative to administration of the test drug is not reasonable OR there is another obvious cause of the AE. (Also entered for a subject with overdose without an associated AE.)

7.7.2.4.3 Reference Safety Information (RSI) for the Assessment of Expectedness of Adverse Events

The Reference Safety Information (RSI) for assessing the expectedness of an adverse event for the investigational product in this current trial is to be the most recent product labeling for ezetimibe.

7.7.2.5 Reporting Safety Observations by the Investigator to the Sponsor

7.7.2.5.1 Expedited Reporting of Safety Observations by the Investigator to the Sponsor

Any occurrence of the following events or outcomes in a subject in the trial must be reported expeditiously by the investigator or qualified designee to the sponsor’s Global Safety representative or designee using the Safety Data Reporting Form 1727 provided by the sponsor/designee **within 1 working day of becoming aware of the event**.

1. SAE (including SAEs associated with pregnancy, exposure during pregnancy or lactation);
2. Death;
3. Planned hospitalizations (not previously reported in the medical history);
4. Closely monitored event;
5. Incidents associated with the device;

6. Cancer.
7. Overdose

Any occurrence of a product quality complaint by a subject in the trial must be reported expeditiously by the investigator or qualified designee to the sponsor or designee using the Research and Commercialization Quality (RCQ) Clinical Complaint/GCP Inquiry Form provided by the sponsor/designee **within 1 working day of becoming aware of the event**.

Any occurrence of the following events or outcomes in a subject in the trial must be reported expeditiously by the investigator or qualified designee to the sponsor or designee using the Safety Data Reporting Form 1727 **within 5 working days of becoming aware of the event**.

1. Pregnancy, exposure during pregnancy or lactation NOT associated with an SAE;

If the investigator is unsure about when to report an observation from the lists above, the event or outcome should be reported to the sponsor or designee using the Safety Data Reporting Form 1727 within 1 working day.

Any observation reported to the sponsor or designee via the Safety Data Reporting Form 1727 that is also an AE, is to be recorded in the CRF (**Section 9.2**), as well as in the subject's source documentation along with any actions taken as a result of AE and follow-up results.

If an autopsy is performed, the de-identified autopsy report must be provided to the sponsor within 1 working day of the results being available.

The Safety Data Reporting Form 1727 requires that the investigator assess causality of the event relative to the investigational product administered in the trial (Causality is described in **Section 7.7.2.4.2**).

7.7.2.5.2 Expedited Reporting by the Sponsor to a Regulatory Health Authority

Global Safety will monitor data for safety. The Sponsor will manage the expedited reporting of relevant safety information to concerned health authorities, competent authorities, and IRBs/IECs in accordance with local laws and regulations.

7.7.2.6 Discontinuation, Treatment Interruption, and Unblinding of Blinded Treatment Due to Safety Observations

7.7.2.6.1 Discontinuation

See **Section 7.3.3** for the criteria by which a subject must be discontinued. Should a subject be discontinued from the trial, complete the visit activities as specified for discontinuation in the Trial Flow Chart in **Section 2.2**.

7.7.2.6.2 Temporary Interruption of Treatment for a Subject

The investigator (sub-investigator) can temporarily discontinue the administration of the study drug if it is judged necessary from the standpoint of safety, such as the onset of an adverse event during treatment.

7.7.2.6.3 Modification of Dose and/or Administration of Investigational Product for a Subject

The dose and administration to any subject may not be modified. If necessary a subject must be discontinued for the reasons described in **Section 7.3.3**.

7.7.2.6.4 Unblinding Treatment for a Subject During the Trial

To assess an occurrence of a safety observation, Global Safety may unblind the treatment of any subject for whom a safety observation was reported investigator to the sponsor as described in **Section 7.7.2.5.1**.

Unblinding by the investigator should occur only in the event of adverse event for which it is necessary to know the trial treatment to determine an appropriate course of therapy for the subject. Sealed envelopes or other appropriate mechanisms for unblinding treatment, corresponding to the individual treatment units, which contain the identification of the investigational product are to be provided to the investigator. If the investigator must identify the treatment assignment of an individual subject, the envelope or other unblinding mechanisms can be opened to reveal the subject's treatment. At the end of the trial, sealed envelopes or other unused unblinding mechanisms must be returned to the sponsor. Envelopes or unblinding mechanisms

that have been opened at the trial site must be returned to the sponsor accompanied by a written explanation of the reason why the blind was broken.

7.8 Criteria for Early Termination of the Trial

There are no prespecified criteria for terminating the trial early.

8. STATISTICAL AND ANALYTICAL PLAN

The plans and procedures for statistical analyses related to this study are outlined below, and a separate statistical analysis plan will not be prepared for this study. Before the study is unblinded, the protocol will be amended appropriately should any important change to the statistical plan be made which may impact the fundamentals of the primary analysis, etc. The reasons and timing of other changes to the planned statistical analyses made after the protocol amendment will be described in the clinical study report. Post-hoc exploratory analyses will also be clearly identified in the clinical study report.

8.1 Person-in-charge of Statistical Analysis and In-house Blinding

The sponsor will be responsible for statistical analysis. This study will be conducted as a double-blind study, with the double-blind phase conducted following the in-house blinding procedures. For the final analysis of the double-blind phase, formal unblinding of the final database will not occur until medical and scientific review is conducted to identify subjects in deviation of the protocol and finalization and completeness of the data are declared.

8.2 Items of Analysis

The items of safety and efficacy analysis to evaluate intra- and inter-group differences are listed below.

8.2.1 Items of Safety Analysis

1) Primary safety analysis

The ezetimibe group will be judged to be non-inferior to the placebo group if the upper limit of the two-sided 95% confidence interval of the between-treatment difference in the amount of change in HbA_{1c} from baseline (Visit 2) to the end of 24 weeks of treatment does not exceed the non-inferiority margin of 0.5%.

2) Supportive items of analysis for the analysis of safety

- The amount of change in glycoalbumin and fasting plasma glucose from baseline (Visit 2) to the end of 24 weeks of treatment will be compared between the ezetimibe and placebo groups, and the point estimate values of the difference and confidence intervals will be calculated to examine the effect of ezetimibe on glucose metabolism.

- The proportion of patients with onset of the adverse event "exacerbation of diabetes" (Decided carefully by investigator considering index of glucose control, diabetic medications, compliance of diet therapy and exercise therapy) will be compared between the ezetimibe and placebo groups to examine the effect of ezetimibe on glucose metabolism.

- The proportion of patients* with changes to diabetes medications due to worsening of diabetes will be compared between the ezetimibe and placebo groups to examine the effect of ezetimibe on glucose metabolism. (However, small changes in insulin dosing +/-5U are excluded)

*: Including subjects having change in diabetic medications due to a trend of worsening of diabetes not judged to be an adverse event or for the the prevention of worsening of diabetes.

3) Other items of analysis for the analysis of safety

All items of for the evaluation of safety will be examined based on clinical evaluation and statistical evaluation. Change from before the start of treatment (Visit 2 and 3) will be examined for the items for the evaluation of safety and tolerability such as laboratory test values and vital signs.

8.2.2 Items of Efficacy Analysis

Secondary items of analysis for efficacy

The percent change in the following items from baseline (Visit 2) to the end of 24 weeks of treatment (Visit 7) will be compared between the ezetimibe and placebo groups, and the point estimate values of the difference and confidence intervals will be calculated to examine the efficacy of ezetimibe on lipid metabolism: LDL-cholesterol, total cholesterol, triglycerides, HDL-cholesterol, and non-HDL-cholesterol.

8.2.3 Derivations of Safety Endpoints

Important predefined limits of change of interest are for ALT, AST and CK. ALT or AST elevations that are considered clinically important are defined as follows:

- Single measurement of greater than or equal to 3 times the upper limit of normal (≥3xULN) (referred to as a single elevation).
- Measurements ≥3xULN observed at 2 or more consecutive visits (referred to as a consecutive elevation).
- Single measurement ≥3xULN which is the last available measurement (referred to as a presumed consecutive elevation).
- Single measurement ≥3xULN during treatment or within 2 days after the end of treatment and followed by a measurement <3xULN which is taken more than 2 days after the last dose of treatment (referred to as a presumed consecutive elevation as well). Note: this situation could arise in the case of early discontinuation of study medication.

For CK, clinically important single elevations are defined as elevations in CK ≥10xULN, elevations in CK ≥10xULN with muscle symptoms and elevations in CK ≥10xULN with drug-related muscle symptoms.

8.3 Data Sets

8.3.1 Data Set for Safety

The data set for the statistical analysis of safety (primary endpoint and supportive endpoints) is the Per Protocol Set (PPS). Missing values will not be replaced with other values. The Full Analysis Set (FAS) will be used for sensitivity analysis of the primary trial objective.

1) Full Analysis Set (FAS)

FAS is defined as all randomized subjects excluding the following:

- Subjects from whom written informed consent for participation in this study was not obtained
- Subjects who have not been diagnosed with type 2 diabetes mellitus

- Subjects who are not undergoing treatment with oral diabetes drugs or insulin
- Subjects who did not receive any study drug during the study period
- Subjects for whom no baseline data (Visit 2) were obtained or if obtained, no subsequent data were obtained

2) Per Protocol Set (PPS)

PPS is defined as all randomized subjects excluding the following (also, data of HbA_{1c}, glycoalbumin and fasting plasma glucose after change in diabetes medication will not be used):

- Subjects excluded from FAS
- Subjects in violation of inclusion or exclusion criteria
- Subjects with a medication rate (days study drug was taken/stipulated days of study drug administration) of less than 75%

2) All subjects treated (AST)

Since the analysis of adverse events and adverse drug reactions should be as comprehensive as possible, all subjects who take at least one dose of the study drug during the treatment period will be included in the population for analysis.

8.3.2 Data Set for Efficacy

The data set for the statistical analysis of efficacy is FAS (please see 8.3.1). Missing values will not be replaced with other values.

8.4 Statistical Procedures

8.4.1 Statistical Methods for Safety

(1) Method of analysis of the primary endpoint and supportive endpoints

1) Primary endpoint (HbA_{1c}) and supportive endpoints of glycoalbumin and fasting plasma glucose

The longitudinal analysis of covariance will be used. In this model, baseline, treatment group, HbA_{1c} (2 categories: less than 7.4%, 7.4% to less than 8.4%), insulin use (2 categories: yes, no), time, “time x treatment group” interaction will be included. So as not to place restriction on the time-course curve of mean values, time will be a categorical variable. Between-group difference in mean amount of change from baseline at measurement time points will be estimated and tested based on this model. Correlation between time-course measured values will be modeled using unstructured covariance matrix, but if calculation does not converge, Toeplitz will be used for correlation.

Based on the above model, the difference in least squares mean from the placebo group and standard error as well as the 95% confidence interval will be calculated. The least squares mean value of the amount of change from baseline with each group and standard error as well as the 95% confidence interval will also be calculated. As sensitivity analysis of the primary endpoint, the same analysis will be conducted with FAS.

2) Other supportive endpoints

Between-group comparison will be conducted using Fisher’s exact test for the proportion of subjects having onset of “exacerbation of diabetes” as an adverse event. Between-group comparison using Fisher’s exact test will also be conducted for the proportion of subjects with changes to diabetes medications due to worsening of diabetes.

(2) Other analytical methods

1) Summary statistics

Summary statistics are the number of patients, mean value, standard error, median value, minimum value, and maximum value. Standard deviation will be calculated in place of standard error for demographic variables and baseline characteristics.

2) Adverse events and adverse drug reactions

The number of subjects with adverse events and the incidence of adverse events will be presented by treatment group. The number and incidence of adverse events by system organ class and event (symptoms and findings) will also be tabulated. For each event, tabulation by severity classification (mild, moderate, severe, life-threatening) will also be conducted. Tabulation will also be conducted of the number and incidence of serious adverse events and significant adverse events.

The same tabulations will be conducted for adverse drug reactions.

If the same symptom occurs in multiple degrees of severity in a single patient, tabulation will be conducted based on the most severe degree of severity.

Also summarized by Tier-approach.

For this protocol, gastrointestinal related AEs, gallbladder-related AEs, allergic reaction or rash AEs, hepatitis-related AEs, single and consecutive elevations in ALT/AST≥3xULN (see Section 8.2.3 for detailed definition), elevations in CK ≥10xULN, elevations in CK ≥10xULN with muscle symptoms and elevations in CK ≥10xULN with drug-related muscle symptoms are considered Tier 1 safety parameters. In addition, exacerbation of diabetes, the broad clinical and laboratory AE categories consisting of the percentage of patients with any AE, a drug related AE, a serious AE, an AE which is both drug-related and serious, and who discontinued due to an AE will be considered Tier 2 endpoints. P-values (Tier 1 only) and 95% confidence intervals (Tier 1 and Tier 2) will be provided for betweentreatment differences in the percentage of patients with events; these analyses will be performed using the Miettinen and Nurminen method (1985), which is an unconditional, asymptotic method.

3) Laboratory test values, vital signs, etc.

1. Tabulation of metric data

For the laboratory test values and measurement values below, summary statistics will be calculated by treatment group and by measurement time point (before treatment to after 24 weeks of treatment). A line graph presenting time-course change of individual patients from before treatment to after 24 weeks of treatment will also be prepared.

- Glucose metabolism (HbA_{1c}, glycoalbumin, fasting plasma glucose)
- Hematology (WBC count, RBC count, hemoglobin, hematocrit, platelet count)
- Biochemistry (AST, ALT, γ-GTP, alkaline phosphatase, LDH, CPK, total bilirubin, direct bilirubin, total protein, BUN, uric acid, creatinine, Na, K, Cl)
- Vital signs (body weight, blood pressure, pulse)

2. Judgment of normal/abnormal

Incidence will be tabulated by treatment group and by measurement time point.

8.4.2 Statistical Methods for Efficacy

For LDL-cholesterol, total cholesterol, triglycerides, HDL-cholesterol, and non-HDL-cholesterol, the percent change from baseline at each measurement time point and summary statistics of measured values will be calculated by treatment group. For the percent change, analysis will be conducted using the same model of longitudinal analysis of covariance used for the primary safety endpoint.

8.4.3 Other analyses

8.4.3.1 Demographic variables and baseline characteristics

Demographic variables and baseline values are defined as indicated below for this study. With the FAS population, summary statistics will be tabulated by treatment group for the variables defined below.

1) Demographic variables and baseline characteristics

Continuous values: Age, body weight, BMI, duration of diabetes

Categorical values: Sex (female, male), age [< 65, ≥ 65 (years)], BMI [< 25, ≥ 25 (kg/m^2)], presence/absence of coexisting diseases, presence/absence of insulin administration.

2) Baseline values

Continuous values: HbA_{1c}, glycoalbumin, fasting plasma glucose

Categorical values: HbA_{1c} (<7.4%, 7.4 to <8.4% [before treatment (Visit 2)])

8.4.3.2 Breakdown of Patients

A table summarizing the patients enrolled in the study will be prepared (i.e., number of randomized patients, number of patients who completed the study, and number of patients who discontinued the study).

The patients who discontinued will be classified by the reason for discontinuation as follows: the onset of an adverse event, the occurrence (discovery) of a major protocol deviation, failure of the patient to return to the hospital, relocation of the patient, withdrawal of consent to participate in the study, and another reason.

8.5 Multiplicity

The level of significance used will be two-sided 5% in all cases. There is only one main hypothesis for this study, and therefore the issue of multiplicity will not occur. No multiplicity adjustment between items of analysis and between time points will be made for other analyses.

8.6 Sample size determination and power

Number of patients: 148 (at least 45 patients, accounting for at least 30% of the 148 patients, will be patients receiving insulin)

[Justification]

The difference between the ezetimibe and placebo groups in least squares mean of the amount of change in HbA_{1c} from baseline (Visit 2) to 24 weeks after treatment is set at 0.1%, with standard deviation of 0.7% and non-inferiority margin of 0.5%. With this hypothesis, the number of patients per group is 66 (total: 132 patients) with two-sided 5% (one-sided 2.5%) probability of type 1 error, 90% power, and 1:1 allocation to the ezetimibe and placebo groups. The sample size was set at a total of 148 patients to take dropouts (10%) into account.

The justification for the difference between ezetimibe and placebo groups in least square mean of 0.1%, standard deviation of 0.7%, and non-inferiority margin of 0.5% used above is presented below.

(1) In the open-label glucose metabolism study with ezetimibe (12-week treatment), the amount of change in HbA_{1c} was 0.15%. Although this study did not have a placebo control group, the amount of change with placebo was assumed to be 0.05%. The difference in least squares mean between the ezetimibe and placebo groups (0.15 – 0.05) was set at 0.1%.

(2) The standard deviation of the amount of change HbA_{1c} after 28 weeks of treatment in diabetic patients was 0.7% in the long-term study of ezetimibe and atorvastatin co-administration.

(3) The clinical index of exacerbation of diabetes of increase in HbA_{1c} of 0.5% was used.

8.7 Sub-group Analysis

The sub-group analyses below will be conducted for exploratory purposes, and only summary statistics by treatment group will be calculated.

- Baseline HbA_{1c} value (<7.4%, 7.4 to <8.4%)

- Insulin administration (Yes, No)

If an important factor is identified during the conduct of the study which may impact the primary endpoint besides the above variables, a review will be conducted under blinded conditions, and if appropriate, including the factor as a covariate in the model will be considered.

8.8 Interim Analysis

No formal interim analysis is planned.

8.9 Handling of Patients

The handling of patients and data will be decided upon discussion with the medical expert.

9. ADHERENCE TO ETHICAL, REGULATORY, AND ADMINISTRATIVE CONSIDERATIONS

The trial must be conducted in accordance with Good Clinical Practice (GCP) as outlined in the International Conference on Harmonisation of Technical Requirements for Registration of Pharmaceuticals for Human Use (ICH) Guidelines, E6 Good Clinical Practice: Consolidated Guidance and other applicable laws and regulations. In addition, the trial must be conducted in accordance with: (i) the USA Code of Federal Regulations (CFR) if the trial is conducted under a USA IND, regardless of the country involved; (ii) the European Union (EU) Clinical Trial Directive (CTD) and local regulations if the trial is conducted in the EU; and (iii) any specific local regulations if the trial is conducted elsewhere.

9.1 Ethical Conduct of the Trial

9.1.1 Independent Ethics Committee or Institutional Review Board

Prior to initiation of the trial at any site, the trial, including the protocol, informed consent, and other trial documents must be approved by an appropriate Institutional Review Board (IRB) or Independent Ethics Committee (IEC). The IRB/IEC must be constituted according to applicable regulatory requirements. As appropriate, amendments to the protocol must also be approved by the IRBs/IECs before implementation at the sites, unless warranted to eliminate an immediate hazard. The IRB/IEC approval should be obtained in writing, clearly identifying the trial, the documents reviewed (including informed consent), and the date of the review. The trial as described in the protocol (or amendment), informed consent, and other trial documentation may be implemented only after all the necessary approvals have

been obtained and the sponsor has confirmed that it is acceptable for the investigator to do so.

In the event that the IRB/IEC requires changes in the protocol, the sponsor shall be advised and must approve the changes prior to implementation. The investigator shall not modify the trial described in the protocol once finalized and after approval by the IRB/IEC without the prior written approval of sponsor.

In countries where the investigator submits the trial protocol and statement of informed consent to the IRB/IEC, the investigator or qualified designee will forward the approvals to the sponsor.

9.1.2 Subject Information and Consent

The details of the protocol must be provided in written format and discussed with each potential subject, and written informed consent must be obtained for all subjects before any trial-related procedure is performed. In obtaining informed consent, the information must be provided in language and terms understandable to the subject. The subject, or the subject's legal representative, must give their written consent to participate in the trial. The signed and dated consent form itself must be retained by the investigator as part of the trial records. A copy of the signed and dated consent form must be given to the subject. The consent form must include all of the required elements of informed consent in accordance with ICH Guidelines E6 and local laws. In addition, the sponsor specifically requests that the consent form identify it as the sponsor and state that use of the investigational product(s) is experimental and the side effects of the investigational product(s)are not completely known. The consent form must be approved by the appropriate IRB/IEC and sponsor before trial initiation at a trial site. Any subsequent changes to the approved informed consent form must be reviewed and approved by the appropriate IRB/IEC and sponsor before implementation.

9.1.3 Subject Identification Card

A Subject Identification Card is provided to each subject to carry on his or her person (eg, in a wallet) at all times while the subject is participating in the trial. The Subject Identification Card must be provided to the subject no later than when IMP is dispensed. The card is to be shown to caregivers in the event of an emergency.

At a minimum, the card must contain the following information:

1. Protocol number;
2. The subject’s protocol identification number;

3. A statement identifying the card-carrier as a participant in a clinical trial (eg, “This person is participating in a clinical research trial.”);
4. A statement indicating the person might be taking an investigational drug (eg, “This person is taking an experimental drug which could have interactions with other medications, or placebo”); and
5. Contact information in the event of an emergency or hospitalization. The contact information on the card is to be the investigator or a designated site contact, rather an contact from within the sponsor;

The cards may also include other trial-specific information to assist with treatment decisions in the event of an emergency, such as types of concomitant therapies that may, or may not be, permitted as part of emergency treatment. As with any other information provided to subjects, the Subject Identification Card must be approved by the IRB/IEC. Monitors will request that Investigators provide Subject Identification Cards to each subject. Investigators will be asked to request that subjects carry the cards with them while they are participating in the trial.

The Investigator/site should collect the cards at the end of the trial and retain them with other clinical trial documents.

9.1.4 Registration of the Trial

The trial will be registered by the sponsor on a publicly accessible database. The results will be disclosed by the sponsor on a publicly accessible database.

9.2 Reporting Trial Data to the Sponsor

9.2.1 Data Collection Forms

The Sponsor will provide the site with data collection forms, be they Case Report Forms (CRF), either in paper format or electronic Case Report Forms (eCRF); diaries; Electronic Data Capture (EDC) screens; or other appropriate data collection forms as the trial requires. The investigator is to provide subject data according to the Sponsor’s instructions, in the designated data collection form, compliant with GCP practices. The Sponsor will also provide the site with instructions for assisting other parties - such as a central laboratory - to collect data. As instructed by the Sponsor, a designated central laboratory may collect data in a database and provide the completed database to sponsor. All data collection forms and the databases from the trial are the exclusive property of sponsor.

The investigator must maintain records and data during the trial in compliance with all applicable legal and regulatory requirements. Each data point must be supported by a source document at the trial site. Any records or documents used as the source of information (called the "subject source data") are to be retained for review by authorized representatives of the sponsor or a regulatory agency.

The investigator will ensure that there are sufficient time, staff, and facilities available for the duration of the trial to conduct and record the trial as described in the protocol and according to all applicable guidances, laws, and regulations.

A CRFs should be completed as soon as possible after the evaluation has occurred. All dates appearing on the sponsor's subject data collection forms for laboratory tests, cultures, and other data collected, must be the dates on which the specimens were obtained, or the procedures performed.

9.2.2 Preparing Case Report Forms for All Subjects

A CRF must be completed for all subjects who have given informed consent. The Sponsor must not collect subject names, initials, or other personal information that is beyond the scope of the trial from any subject. Subjects are not to be identified by name or initials on the CRF or any trial documents. The only acceptable identification for a subject who may appear on a CRF or trial document is the unique subject identification number. The investigator must maintain contact information for each participant so that all can be quickly contacted by the investigator, if necessary.

All entries into CRFs are the responsibility of the investigator and must be completed by the investigator or a qualified designee. The investigator will acknowledge in writing that he/she has verified the accuracy of the recorded data.

9.2.3 Preparing Case Report Forms for Subjects Who Fail Screening

Data are to be collected from the time the informed consent form is signed until the subject is determined to have failed screening. A CRF with a minimum of the following information must be completed for subjects who fail screening:
(1) demographics, (2) subject status, (3) reason for screen failure, and (4) serious adverse events.

9.3 Publications and Other Rights

9.3.1 Rights to Publish by the Investigator

The investigator has the right to publish or publicly present the results of the trial in accordance with this **Section 9.3** of the protocol. In the event that the protocol is a part of a multi-site trial, it is understood that it is the intent of the sponsor and the investigator to initially only publish or present the trial results together with the other sites, unless specific written permission is obtained in advance from the sponsor to publish separate results. The sponsor shall advise as to the implications of timing of any publication in the event clinical trials are still in progress at sites other than the investigator's site.

The investigator agrees not to publish or publicly present any interim results of the trial without the prior written consent of the sponsor. The investigator further agrees to provide to the sponsor 45 days prior to submission for publication or presentation, review copies of abstracts or manuscripts for publication (including, without limitation, slides and texts of oral or other public presentations and texts of any transmission through any electronic media, eg, any computer access system such as the Internet, World Wide Web, etc) that report any results of the trial. The sponsor shall have the right to review and comment with respect to publications, abstracts, slides, and manuscripts and the right to review and comment on the data analysis and presentation with regard to the following concerns:

1. Proprietary information that is protected by the provisions contained in **Section 9.3.2**;
2. The accuracy of the information contained in the publication; and
3. To ensure that the presentation is fairly balanced and in compliance with FDA regulations.

If the parties disagree concerning the appropriateness of the data analysis and presentation, and/or confidentiality of the sponsor's confidential information, investigator agrees to meet with the sponsor's representatives at the clinical trial site or as otherwise agreed, prior to submission for publication, for the purpose of making good faith efforts to discuss and resolve any such issues or disagreement.

9.3.2 Use of Proprietary or Confidential Information in a Publication

No publication or manuscript shall contain any trade secret information of the sponsor or any proprietary or confidential information of the sponsor and shall be

confined to new discoveries and interpretations of scientific fact. If the sponsor believes there is patentable subject matter contained in any publication or manuscript submitted for review, the sponsor shall promptly identify such subject matter to investigator. If sponsor requests and at sponsor's expense, investigator shall use its best efforts to assist sponsor to file a patent application covering such subject matter with the USA Patent and Trademark Office or through the Patent Cooperation Treaty prior to any publication.

9.3.3 Use of Trial Information in a Publication

Investigator is granted the right subject to the provisions of this protocol to use the results of all work provided by investigator under this protocol, including but not limited to, the results of tests and any raw data and statistical data generated for investigator's own teaching, research, and publication purposes only. Investigator/Institution agrees, on behalf of itself and its employees, officers, trustees, and agents, not to cause said results to be knowingly used for any commercial purpose whatsoever except as authorized by the sponsor in writing.

9.3.4 Authorship of Publications

Authors of publications must meet the International Committee of Medical Journal Editors (ICMJE) guidelines for authorship and must satisfy the 3 criteria that follow:

1. Authors must make substantial contributions to the conception and design of the trial, acquisition of data, or analysis of data and interpretation of results;
2. Authors must draft the publication or, during draft review, provide contributions (data analysis, interpretation, or other important intellectual content) leading to significant revision of the manuscript with agreement by the other authors;
3. Authors must provide written approval of the final draft version of the publication prior to submission.

All contributors who do not meet the 3 criteria for authorship should be listed in an acknowledgments section within the publication, if allowed by the journal, per the ICMJE guidelines for acknowledgment.

9.4 Trial Documents and Records Retention

During the trial and after termination of the trial – including after early termination of the trial – the investigator must maintain copies of all documents and records relating to the conduct of the trial. This documentation includes, but is not limited to, protocols, CRFs and other data collection forms, advertising for subject participation, adverse event reports, subject source data, correspondence with health authorities and IRBs/IECs, consent forms, investigator's curricula vitae/biosketch, monitor visit logs, laboratory reference ranges, and laboratory certification or quality control procedures and laboratory director curriculum vitae. Subject files and other source data must be kept for the maximum period of time permitted by the hospital, institution or private practice, or as specified below. The sponsor must be consulted if the investigator wishes to assign the files to someone else, remove them to another location, or is unable to retain them for the specified period.

The investigator must retain trial records for the amount of time specified by applicable laws and regulations. At a minimum, trial records must be retained for the amount of time specified by ICH Guidelines, the EU Good Clinical Practices Directive, or applicable local laws, whichever is longer:

1. The ICH Guidelines specify that records must be retained for a minimum of 2 years after a marketing application for the indication is approved (or not approved) or 2 years after notifying the appropriate regulatory agency that an investigation is discontinued.
2. The European Union (EU) Commission Directive 2003/63/EC which requires that Essential Documents (including Case Report Forms) other than subjects' medical files, are retained for at least fifteen (15) years after completion or discontinuation of the trial, as defined in the protocol.

All trial documents shall be made available if required by relevant health authorities. The investigator should consult with the sponsor prior to discarding trial and/or subject files.

Sponsor will retain all sponsor-required documentation pertaining to the trial for the lifetime of the investigational product. Archived data may be held on microfiche or electronic record, provided that a back-up exists and that a paper copy can be obtained from it, if required.

10. INVESTIGATORS AND TRIAL ADMINISTRATIVE STRUCTURE

10.1 Sponsor

The sponsor of this trial is indicated in **Section 1**, Title Page.

The trial administrative structure is described in a separately prepared accompanying sheet.

10.2 Investigators

10.2.1 Selecting Investigators

Only investigators qualified by training and experience to perform a clinical investigation with ezetimibe are selected. The sponsor will contact and select all investigators (ie, the legally responsible party[ies] at each trial site), who, in turn, will select their staff.

Investigators and sites are provided on a separately prepared accompanying sheet.

10.2.2 Financial Disclosure Requirement

In connection with the clinical trial described in the protocol, the investigator certifies that, if asked, the investigator will read and answer the Certification/Disclosure Form or equivalent document truthfully and to the best of investigator's ability. Investigator also certifies that, if asked, the investigator will have any other applicable party(ies) (eg, subinvestigators) read and answer the Certification/Disclosure Form as a condition of their participation in the trial.

If the financial interests reported on the Certification/Disclosure Form change during the course of the trial or within 1 year after the last subject has completed the trial as specified in the protocol, the investigator and the other applicable party(ies) are obligated to inform the sponsor of such financial change.

10.2.3 Clinical Study Report Coordinator Investigator

A Clinical Study Report (CSR) will be prepared by the sponsor or its qualified designee to describe the results of the trial. One of the investigators shall be selected by the sponsor to review the CSR and provide approval of the final CSR in

writing. The investigator chosen to review and approve the CSR is to be called the CSR Coordinating Investigator. A second investigator shall be selected as the Alternate CSR Coordinating Investigator. The Alternate CSR Coordinating Investigator is to review and approve the CSR should the first CSR Coordinating Investigator be unable to do so. The sponsor is to select the CSR Coordinating Investigator and Alternate CSR Coordinating Investigator from the investigators using the following criteria:

1. Must be the Principal Investigator at a trial site actively enrolling subjects and participating in the trial;
2. Must be willing and capable of completing the necessary reviews and providing approval of the CSR in writing;

10.3 Central Organizations

Central organizations to be used in the conduct, monitoring, and/or evaluation of this trial (Name and address of the central organizations and other information can be found in a separately prepared accompanying sheet):

- the central laboratory
- the allocation controller
- the Registration Center
- the Emergency Center
- Clinical Research Organization

11. REFERENCES

1. Hsueh, W.A., Law R.E. Cardiovascular risk continuum: implications of insulin resistance and diabetes. Am J Med, 105:4S-14S, 1998.

2. The Japan Atherosclerosis Society. Guideline for prevention of arteriosclerotic diseases. 2007 version.

3. Assmann,G., Schulte, H. The prospective cardiovascular munster (PROCAM) study, Prevalence of hypertension and/or diabetes mellitus and the relationship to coronary heart disease. Am Heart J, 116(6):1713-1724,1988.

4. Garcia-Calvo M, Lisnock J, Bull HG, Hawes BE, Burnett DA, Braun MP, et al. The target of ezetimibe is Niemann-Pick C1-Like 1 (NPC1L1). Proc Natl Acad Sci USA. 2005;102:8132-7.

5. Hawes BE, O'neill KA, Yao X, Crona JH, Davis HR Jr, Graziano MP, et al. In vivo responsiveness to ezetimibe correlates with niemann-pick C1 like-1 (NPC1L1) binding affinity: Comparison of multiple species NPC1L1 orthologs. Mol Pharmacol. 2007;71:19-29.

6. Matsushima T, Saito Y, Yamada N. et al. Clinical condition and differential diagnosis, primary hypercholesterolemia. Nankoudo. 1999:77-80.

7. Kiyono H, Yamamoto A. Clinical medications. 2007;23(6). 571-588.

8. Carleton RA, Dwyer J, Finberg L, Flora J, Goodman DS, Grundy SM, et al. Report of the Expert Panel on Population Strategies for Blood Cholesterol Reduction. A statement from the National Cholesterol Education Program, National Heart, Lung, and Blood Institute, National Institutes of Health. Circulation. 1991;83:2154-232.

9. National Cholesterol Education Program. Second Report of the Expert Panel on Detection, Evaluation, and Treatment of High Blood Cholesterol in Adults (Adult Treatment Panel II). Circulation. 1994;89:1333-445.

10. Guideline for clinical evaluations criteria of antilipemic: PAB/PCD Notification No.1， 1988.

11. Diabetes Treatment Guide
